# Folate Acts in *E. coli* to Accelerate *C. elegans* Aging Independently of Bacterial Biosynthesis

**DOI:** 10.1016/j.celrep.2016.01.051

**Published:** 2016-02-11

**Authors:** Bhupinder Virk, Jie Jia, Claire A. Maynard, Adelaide Raimundo, Jolien Lefebvre, Shane A. Richards, Natalia Chetina, Yen Liang, Noel Helliwell, Marta Cipinska, David Weinkove

**Affiliations:** 1 School of Biological and Biomedical Sciences, Durham University, South Road, Durham DH1 3LE, UK; 2 Department of Nutrition, Shanghai Jiao Tong University School of Medicine, Shanghai 200025, China; 3 Key Laboratory of Pediatric Gastroenterology and Nutrition, Shanghai Institute for Pediatric Research, Shanghai 200092, China; 4 Department of Clinical Nutrition, Xin Hua Hospital affiliated to SJTU School of Medicine, Shanghai 200092, China; 5 Department HIVB, VIVES, Wilgenstraat 32, 8800 Roeselare, Belgium; 6 Biophysical Sciences Institute, Durham University, South Road, Durham DH1 3LE, UK

## Abstract

Folates are cofactors for biosynthetic enzymes in all eukaryotic and prokaryotic cells. Animals cannot synthesize folate and must acquire it from their diet or microbiota. Previously, we showed that inhibiting *E. coli* folate synthesis increases *C. elegans* lifespan. Here, we show that restriction or supplementation of *C. elegans* folate does not influence lifespan. Thus, folate is required in *E. coli* to shorten worm lifespan. Bacterial proliferation in the intestine has been proposed as a mechanism for the life-shortening influence of *E. coli*. However, we found no correlation between *C. elegans* survival and bacterial growth in a screen of 1,000+ *E. coli* deletion mutants. Nine mutants increased worm lifespan robustly, suggesting specific gene regulation is required for the life-shortening activity of *E. coli*. Disrupting the biosynthetic folate cycle did not increase lifespan. Thus, folate acts through a growth-independent route in *E. coli* to accelerate animal aging.

## Highlights

• Limiting folate in *E. coli*, not in *C. elegans*, increases worm lifespan
• An *E. coli* screen for worm longevity identifies folate synthesis as a target
• Folate synthesis influences *E. coli* physiology independently of growth
• Bacterial folate synthesis may be a sustainable target for chronic disease

## Graphical Abstract

**Figure:**
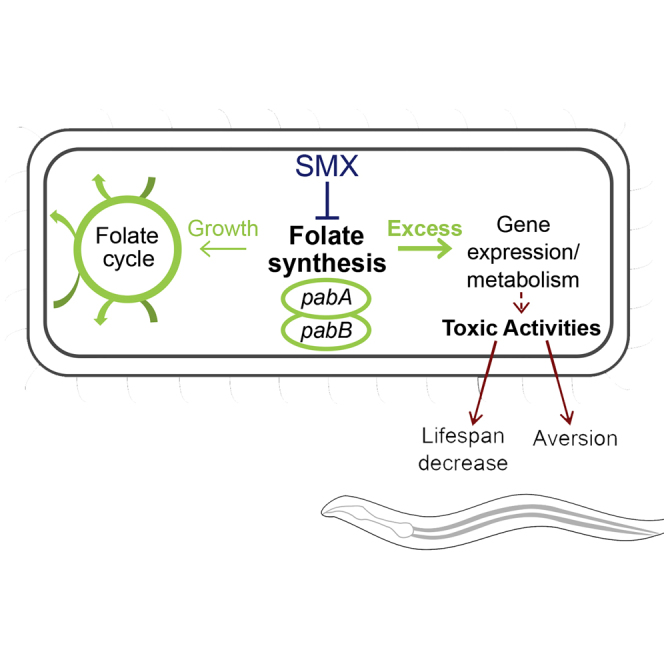

## Teaser

Virk et al. show that inhibiting *E. coli* folate synthesis does not increase *C. elegans* lifespan through changes to *C. elegans* folate, but by acting through an *E. coli* activity. They find nine *E. coli* mutants that extend lifespan. The gene identities suggest that mild bacterial toxicities accelerate host aging.

## Introduction

Both nutrition and the host-associated microbiota are thought to impact longevity (Heintz and Mair, 2014, Rizza et al., 2014). Diet influences the metabolism of gut microbes, which in turn can synthesize nutrients for the host. These interactions make it difficult to unravel the contributions of diet and the gut microbiota to long-term health (Lozupone et al., 2012). This complexity can be addressed with model systems such as the nematode *Caenorhabditis elegans* (Collins et al., 2008). Yet even here, there are numerous interactions between the nutrient agar medium, the *Escherichia coli* bacterial lawn, and the worm. Chemical manipulations of the medium and genetic manipulations of both *E. coli* and *C. elegans* provide tools to understand these interactions (Weinkove, 2015).

Folates in their reduced tetrahydrofolate (THF) form are required as enzymatic cofactors in the folate cycle; a series of metabolic steps found in all cells (including both bacteria and animals) required for cell biosynthesis. Products include purines, pyrimidines, glycine, and methionine, which are required to generate the methyl donor molecule S-adenosyl methionine (SAM) (Bailey and Gregory, 1999). Animals cannot synthesize folates and so obtain folates from their diets and associated microbes (Asrar and O’Connor, 2005, Lakoff et al., 2014). Our previous research showed that *C. elegans* lifespan is increased when *E. coli* folate synthesis is disrupted either by a mutation in the gene *aroD*, which is needed to make aromatic compounds including the folate precursor para-aminobenzoic acid (PABA), or by sulfamethoxazole (SMX), a sulfonamide drug that competes with PABA for the active site of the enzyme dihydropteroate synthase (Virk et al., 2012). This enzyme is a key step in folate biosynthesis and is absent from animals. *C. elegans* obtains folates from *E. coli* and thus several possible mechanisms might explain why *E. coli* folate synthesis affects *C. elegans* lifespan. Distinguishing the effects of folates in bacteria and folates in their animal hosts is important because folate supplementation is beneficial to human health and any intervention would need to maintain healthy levels of serum folate.

Dietary, or caloric, restriction has been shown to extend the lifespan of *C. elegans* (Greer and Brunet, 2009, Mair and Dillin, 2008). SMX does not slow *E. coli* growth and therefore has no effect on food availability. Furthermore, *C. elegans* grow and reproduce normally (Virk et al., 2012). Thus, a limitation of macronutrients is an unlikely explanation. Alternatively, inhibition of *E. coli* folate synthesis may influence *C. elegans* lifespan by limiting dietary folate and/or a specific change in folate-dependent nutrients (Lee et al., 2015). For example, restriction of methionine increases lifespan in rodents and influences lifespan in *Drosophila* (Grandison et al., 2009, Sanchez-Roman and Barja, 2013). Mutation of *C. elegans sams-1,* the gene encoding SAM synthase, extends lifespan (Hansen et al., 2005). The diabetes drug metformin increases *C. elegans* lifespan in a manner dependent on the *E. coli* strain and changes in *C. elegans* folate and methionine metabolism are implicated in mediating the lifespan extension (Cabreiro et al., 2013).

Another possible explanation is that folate synthesis inhibition increases *C. elegans* lifespan by altering *E. coli* physiology. *E. coli* can accumulate in the intestine of older *C. elegans* adults and because treatment of *E. coli* with antibiotics or UV increases worm lifespan, this accumulation is widely thought to accelerate *C. elegans* aging (Garigan et al., 2002, Gems and Riddle, 2000, McGee et al., 2011). More subtly, changes in bacterial toxicity caused by changes in bacterial metabolism might influence *C. elegans* aging. The *E. coli ubiG* mutant, which cannot synthesis coenzyme Q/ubiquinone, increases worm lifespan by influencing bacterial respiration rather than dietary intake of Q (Saiki et al., 2008).

Here, we show that modulating folate uptake or the folate cycle in *C. elegans* does not affect lifespan, suggesting *E. coli* folate influences *C. elegans* lifespan by acting on *E. coli* physiology. Apart from the Q synthesis genes and *aroD* (Saiki et al., 2008, Virk et al., 2012), little is known about how *E. coli* genetics influences *C. elegans* lifespan. A genetic screen of over 1,000 *E. coli* mutants shows that bacterial growth does not correlate with *C. elegans* survival and only a few specific interventions increase *C. elegans* lifespan, including the mutation of genes involved in *E. coli* folate synthesis, but not in the *E. coli* folate cycle. In addition to its role in bacterial growth, we propose that folate acts to change *E. coli* physiology in a way that accelerates *C. elegans* aging.

## Results

### Genetic Disruption of *C. elegans* Folate Uptake and Restoration by Supplementation

**Figure 1:**
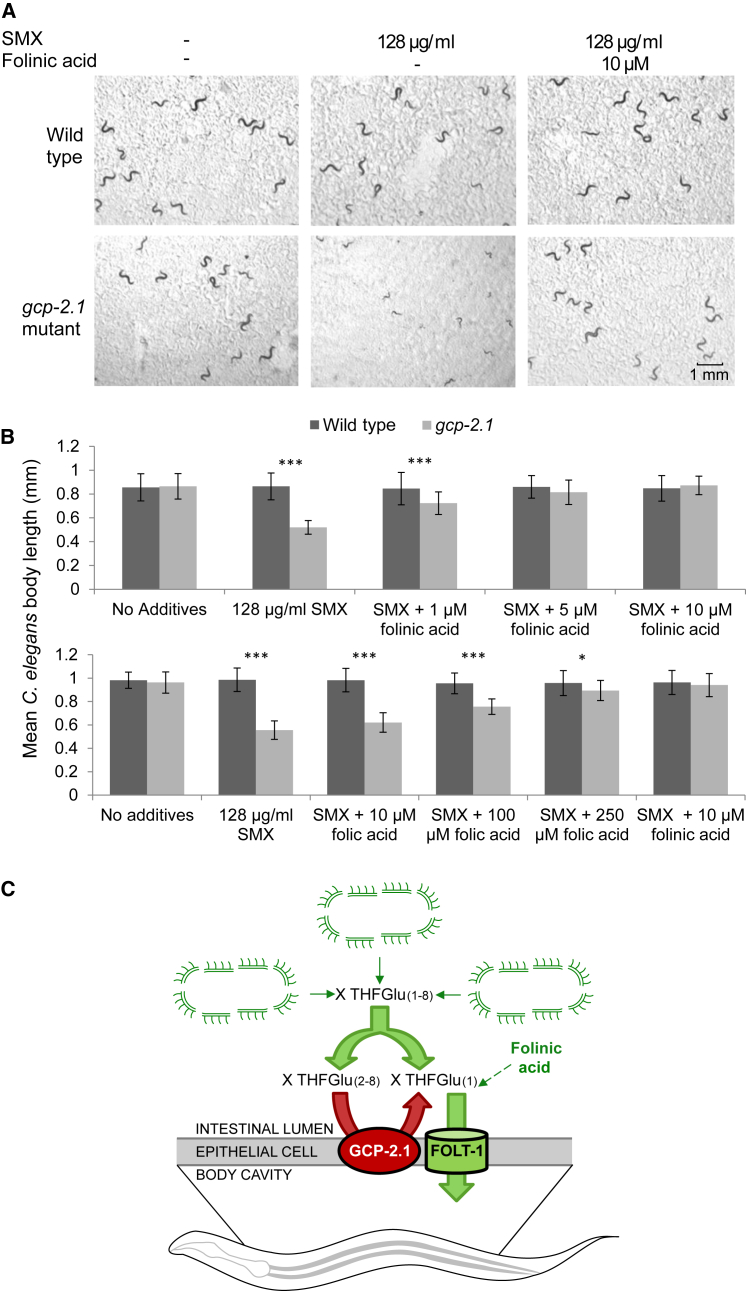
The *C. elegans gcp-2.1* Folate Uptake Mutant Is Sensitive to SMX-Treated *E. coli* and Is Rescued by Folinic Acid (A) *gcp-2.1* mutants develop as wild-type worms on untreated OP50 *E. coli*, but SMX treatment delays the growth of *gcp-2.1* mutants, and this defect can be rescued by 10 μM folinic acid. The images were taken after 48 hr of growth at 25°C. (B) Quantification of the growth, as measured by body length. The error bars represent SD. The *gcp-2.1* mutant growth on SMX is restored at 5 μM and 10 μM folinic acid and partially with 1 μM folinic acid (^∗^p < 0.05, ^∗∗^p < 0.01, and ^∗∗∗^p < 0.005) (t test). Folic acid can fully rescue growth only at 250 μM. (C) Model showing that *E. coli* folate synthesis generates THFs with up to eight glutamate residues and various one carbon groups, xTHFGlu_1–8_ (x = methyl, formyl, methenyl, and methylene). The *C. elegans* GCPII GCP-2.1 cleaves glutamate residues from xTHFGlu_2–8_ to generate xTHFGlu_1_, the preferred substrates of the *C. elegans* reduced folate transporter FOLT-1. Folinic acid, as an xTHFGlu_1_ (5-formyl THF), can be taken up directly by FOLT-1, bypassing GCP-2.1.

To distinguish the effects of *C. elegans* folate from *E. coli* folate, we targeted folate uptake in *C. elegans*. The reduced folate carrier FOLT-1 takes up THFs across the intestinal epithelial membrane (Balamurugan et al., 2007). The published *folt-1* deletion allele causes sterility, so we turned to another *C. elegans* mutant predicted to disrupt folate uptake. In mammals, glutamate carboxypeptidase II (GCPII) cleaves glutamates from polyglutamated THFs in the gut, to create monoglutamated folates that are preferentially imported by folate carriers and transporters (Halsted et al., 1998). The *E. coli* diet contains predominantly polyglutamated THFs (Kwon et al., 2008, Virk et al., 2012), suggesting that *C. elegans* requires GCPII activity. There are three *C. elegans* genes that encode a GCPII homolog (Supplemental Information). Animals lacking the GCPII gene *gcp-2.1*, *WB Gene*: WBGene00020082, appear healthy and develop normally on *E. coli* OP50, but develop slowly and are uncoordinated and sterile on OP50 treated with 128 μg/ml SMX (Figure 1A). This phenotype is rescued completely by supplementation with 10 μM formyl THF monoglutamate, a naturally occurring reduced folate also known as folinic acid or leucovorin (Figures 1A and 1B). Folinic acid can rescue the *gcp-2.1* phenotype at a 20-fold lower concentration than can folic acid, the oxidized folate used commonly in dietary supplements (Figure 1B). These results are consistent with the specificity of FOLT-1 for reduced folates (Balamurugan et al., 2007) and a role for GCP-2.1 in folate uptake (Figure 1C).

*E. coli* does not have folate uptake transporters, but can synthesize folate from the folate breakdown products PABA, which can freely diffuse through membranes, and PAB-Glutamate, which is taken up by an active transport system (Hussein et al., 1998). Thus, folic acid restores folate synthesis in an *aroD* mutant, most clearly at concentrations of 100 μM or more (Virk et al., 2012). In the presence of SMX, which competes with PABA for the enzyme dihydropteroate synthase, it is likely that more folate breakdown products would be required to restore folate synthesis. For example, PABA is required at a concentration of 250 μM to fully reverse the lifespan extension caused by 505 μM (128 μg/ml) SMX (Virk et al., 2012). It is unlikely that 10 μM folinic acid would provide enough breakdown products to restore *E. coli* folate synthesis in competition with 505 μM SMX. Thus, we conclude that folinic acid rescues *gcp-2.1* by directly supplementing *C. elegans* folate (Figure 1C).

### Modulation of *C. elegans* Folate Status Does Not Influence Lifespan

**Figure 2:**
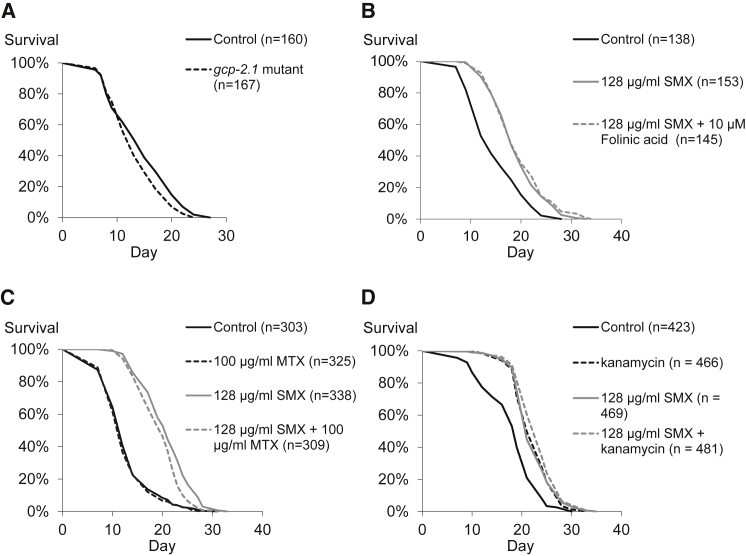
Lifespan Analyses of Perturbations to *C. elegans* Folate and Comparison of SMX and Kanamycin Treatment (A) The *gcp-2.1(ok1004)* mutant does not increase lifespan. (B) 10 μM folinic acid does not affect the lifespan extension caused by 128 μg/ml SMX. (C) MTX has no effect on *C. elegans* lifespan. (D) Kanamycin and SMX have a very similar effect on *C. elegans* lifespan. See Table S1 for lifespan conditions and statistics.

Turning to effects on aging, we found that the *C. elegans gcp-2.1* mutant has a similar, if not slightly shorter lifespan, than wild-type controls (p = 0.0227; Figure 2A). Furthermore, the increased *C. elegans* lifespan caused by SMX was unaffected by supplementation with 10 μM folinic acid (Figure 2B). Methotrexate (MTX), a dihydrofolate reductase inhibitor specific to animals, inhibits the *C. elegans* folate cycle. 100 μg/ml MTX causes developmental defects in *nuc-1* mutants and is five times the concentration required to cause this phenotype (Mello et al., 1991, Virk et al., 2012). MTX did not affect the lifespan of *C. elegans* and also failed to influence the lifespan of worms on SMX-treated bacteria (Figure 2C). Together, these results suggest that the SMX-induced lifespan increase cannot be explained by decreased *C. elegans* folate uptake or impaired folate-dependent *C. elegans* metabolism.

### SMX and Kanamycin Treatment Cause an Identical Increase in *C. elegans* Lifespan

Treating *E. coli* with ultraviolet irradiation or antibiotics increases *C. elegans* lifespan, suggesting that *E. coli* possess a life-shortening activity (Garigan et al., 2002, Gems and Riddle, 2000). We compared OP50 treated with SMX, which does not influence *E. coli* viability, with OP50 treated with kanamycin, a bacterial translation inhibitor. Addition of kanamycin to the bacterial lawn stops cells forming further colonies (Virk et al., 2012). Using large cohorts, worms maintained on kanamycin-treated bacteria showed an almost identical survival curve to those on bacteria treated with SMX (25% increase in mean lifespan compared to wild-type, p = < 0.0001), with a small further increase in lifespan (4%, p = 0.0008) when both drugs were combined (Figure 2D). While there are many possible explanations for this result and SMX and kanamycin have very different targets and effects on *E. coli* metabolism, the lack of a substantial additive effect suggests that both drugs might inhibit a shared downstream process that shortens *C. elegans* lifespan.

### It Is Unlikely that *E. coli* Shortens Lifespan Solely through Intestinal Accumulation

**Figure 3:**
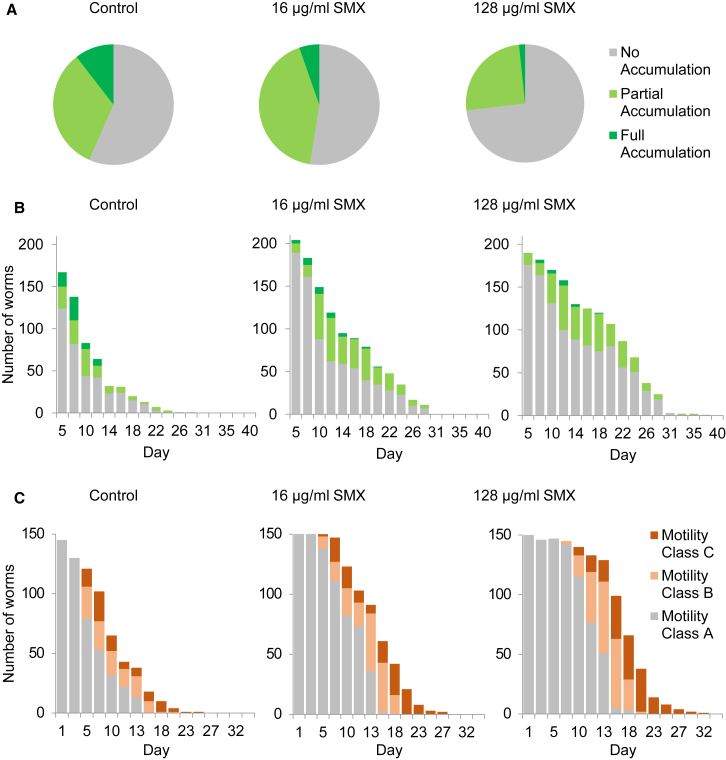
Intestinal Accumulation of Bacteria Does Not Occur in All Animals and Is Not Prevented by SMX (A) Accumulation in recently dead animals as assessed by visualizing *E. coli* GFP in the intestinal lumen. The data are pooled from two biological replicates. (B) Numbers of alive worms at indicated time points with classification of accumulation. (C) Motility analysis of *glp-4(bn2)* worms on OP50 treated with 0, 16 μg/ml, and 128 μg/ml SMX. Each worm was scored as belonging to motility class A (constantly moving), B (moves when prodded), or C (twitches only) as described (Herndon et al., 2002).

Kanamycin and similar treatments are thought to increase *C. elegans* lifespan by preventing *E. coli* accumulation in the intestinal lumen (Garigan et al., 2002). To test whether SMX prevents accumulation, we performed lifespan experiments with worms maintained on *E. coli* OP50 expressing GFP. These fluorescent bacteria could be observed accumulating in live worms from the beginning of adulthood through to death. A long pass green filter, which allows red light through, was used to distinguish GFP-expressing *E. coli* from gut autofluorescence (Figure S1). This distinction is harder to make using the narrow band green filters employed in most studies of GFP-expressing bacteria. We detected accumulation of bacteria in some worms from day 5 of adulthood onward. However, many worms did not appear to accumulate bacteria at all. Approximately 50% of recently dead worms showed no visible accumulation (Figure 3A), suggesting that bacterial accumulation did not contribute to their death. This heterogeneity in accumulation is missed when groups of worms rather than individuals are assessed (Gomez et al., 2012, Portal-Celhay et al., 2012). SMX decreased the number of worms that died with bacterial accumulation, but did not prevent accumulation from occurring in all animals. Across the whole lifespan, SMX delayed the onset of bacterial accumulation, but did not prevent it (Figure 3B). SMX causes the GFP expressing OP50 *E. coli* to appear brighter than in untreated conditions, confirming that these bacteria are capable of producing increased protein. SMX prolonged the time that *C. elegans* stay mobile, consistent with a decrease in the rate of aging (Figure 3C). The structure and function of the *C. elegans* intestine declines with age. Thus, like motility, bacteria accumulation is a biomarker, but not necessarily a cause, of aging.

### *E. coli* Mutants Influence *C. elegans* Lifespan Independently of *E. coli* Growth

To further understand how *E. coli* influence *C. elegans* lifespan, we conducted a screen of over 1,000 *E. coli* K12 mutants from the Keio collection (Baba et al., 2006). We tested deletions in all non-essential genes predicted to encode enzymes in the folate cycle or related pathways (25 genes; Table S2) and deletions of 981 randomly selected genes of known function (Experimental Procedures; Table S2). Scoring across the lifespan is impractical for a large-scale screen, so we scored survival at a single time point (day 11/12) close to the median lifespan. This strategy allows greater statistical power than scoring near the end of the survival curve and the identification of strains that shorten, as well as extend, *C. elegans* lifespan.

**Figure 4:**
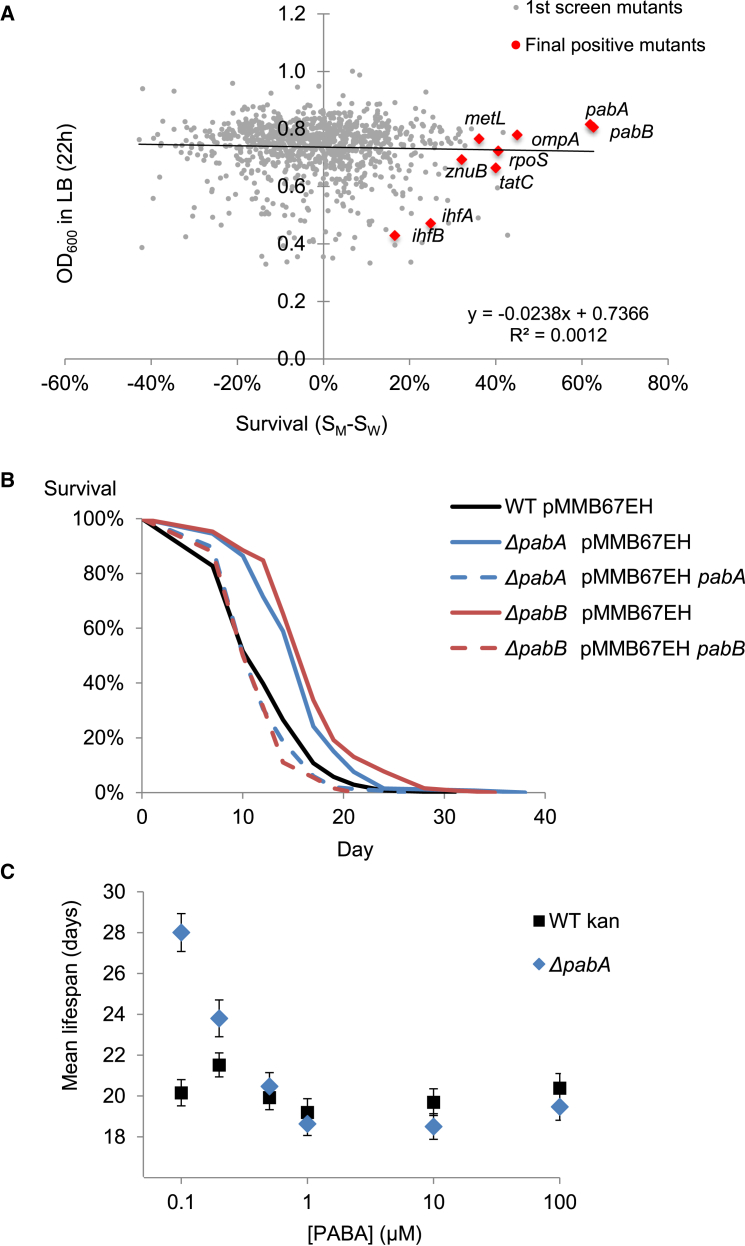
A Screen of *E. coli* Deletion Mutants for *C. elegans* Survival Identified Nine Mutants that Robustly Extend Lifespan (A) No correlation between growth of mutant strains in LB (Baba et al., 2006) and normalized *C. elegans* survival at day 11 or 12. The mutants that increase lifespan after the fourth round of the screen are indicated. (B) *pabA* and *pabB* mutants increase *C. elegans* lifespan, and this increase is reversed by gene complementation. See Table S1 for details. (C) Mean lifespan (with SD) at various concentrations of PABA on defined media plates, comparing worms on the *E. coli pabA* mutant with worms on WT *E. coli*. At 0.1 and 0.2 μM PABA, the lifespans on *pabA* and wild-type *E. coli* are significantly different (see Results and Table S1 for details). The error bars represent SD.

The mutants were scored in batches. Each batch contained several mutants and three wild-type strains. The distribution in survival shown by these controls did not differ from the distribution across all mutants tested, suggesting that mutating single *E. coli* genes had no detectable large-scale effect on *C. elegans* lifespan (Supplemental Information; Figure S2). To account for batch-to-batch variation, we subtracted the mean survival of the wild-type in a batch, S_W_, from the survival of each mutant S_M_ in that batch (Supplemental Information). S_M_ - S_W_ revealed a narrower distribution and a set of candidates for strains that increased *C. elegans* lifespan (Figure S2). Using growth data from Baba et al. (2006), we found no correlation between strain growth and survival of *C. elegans* (Figure 4A), suggesting that *E. coli* growth rate does not influence *C. elegans* aging.

### Nine *E. coli* Mutants Robustly Increase *C. elegans* Lifespan

To be confident of identifying individual mutants that increased *C. elegans* lifespan, we repeated the screen for the 67 strains that caused worms to survive at least 15% more than on the control strain. We undertook full lifespan analysis of the 22 strains that passed this second round. Finally, we retested the 11 strains that passed this third round and included full lifespan analysis of the strains with the mutation complemented by the wild-type *E. coli* gene. This step ruled out lifespan increases from spontaneous second-site mutations (Supplemental Information). There were nine deletions that passed this final test, representing less than 1% of genes tested.

Three of the identified mutants (*metL*, *ihfA*, and *ihfB*) caused the bacterial lawn to appear more liquid than normal. This environment would have a strong influence on *C. elegans* physiology (Lewis and Fleming, 1995). The other mutants discovered had no visible effect on the *E. coli* lawn and so are more likely to influence lifespan through biological, rather than physio-chemical mechanisms. These mutants included a deletion of *rpoS*, a stationary phase sigma factor. This transcription factor regulates over 200 genes in response to low nutrients or other stresses (Battesti et al., 2011). Other deletions that extended lifespan included *tatC*, a gene encoding a component of the twin arginine translocation pathway, which transports folded proteins to the periplasm (Stanley et al., 2001), *ompA*, an abundant constituent of the outer membrane (Smith et al., 2007), and *znuB*, which encodes part of the ZnuABC zinc transporter, needed to take up zinc at low concentrations (Patzer and Hantke, 1998). Finally, we isolated deletions in *pabA* and *pabB* (Figure 4B). These genes encode two enzymes that associate and catalyze key steps in PABA synthesis (Green et al., 1996). In summary, the screen underlined the importance of *E. coli* folate synthesis in *C. elegans* lifespan regulation and identified other genes with diverse functions.

Apart from the *pabA* and *pabB* mutants, the mutants grow slower on the petri dish than the wild-type strain (Figure S3A). There was a positive, but not significant, correlation between extent of the lifespan extension and the growth rate of the strain (Figure S3B). We noticed that *C. elegans* spent more time on the bacterial lawn if the *E. coli* strain was one of the life-extending mutants or had been treated with SMX (Figure S3C). SMX treatment of OP50 also produced a similar decrease in aversion. Thus, the increased lifespan of *C. elegans* is not caused by decreased exposure to food or to *E. coli*. *C. elegans* avoids toxic bacteria, and this avoidance is thought to be triggered by perception of disruption to worm metabolism caused by bacterial toxins (Melo and Ruvkun, 2012). Thus, the mutations isolated in the screen, or chemical inhibition of folate synthesis, may remove toxicity from *E. coli.*

### Disruption of the *E. coli* Folate Cycle Does Not Increase *C. elegans* Lifespan

Although the identification of *pabA* and *pabB* was consistent with *E. coli* folate synthesis inhibition increasing *C. elegans* lifespan, we were surprised that no genes involved in the folate cycle or related pathways were identified. An exception was *metL*, which encodes an enzyme needed in two early stages of methionine biosynthesis. However, deletions in other methionine synthesis genes did not have a lifespan phenotype. Because of the variation found in the screen, we wanted to make sure that we had not missed any folate-related genes. We performed full lifespan analysis on *E. coli* mutants in 23 non-essential genes involved in the folate cycle or related metabolic pathways. None of these mutants extended lifespan apart from a small, but significant, effect of a deletion of *glyA* (Table S1). Thus *E. coli* folate synthesis, but not the folate cycle, which is needed for *E. coli* growth, limits *C. elegans* lifespan.

### *C. elegans* Lifespan Can Be Altered through the Availability of PABA to *E. coli*

Folate synthesis is essential for *E. coli* growth because *E. coli* cannot take up intact folate. However, SMX or mutation of *pabA* or *pabB* do not decrease *E. coli* growth rate under the conditions that they increase *C. elegans* lifespan (Virk et al., 2012) (Figure S3A). Thus, these interventions must only remove folate in excess of that required for *E. coli* growth. To understand the relative levels at which folate synthesis is required to limit lifespan compared to requirements for growth, we took advantage of the fact that PABA synthesis in *E. coli* can be bypassed by adding exogenous PABA, which can diffuse across membranes and that *C. elegans* cannot make folate from PABA. The growth of *pabA* and *pabB* mutants on peptone-based NGM, suggests this medium contains a source of PABA. To remove this PABA while minimizing changes to the nutritional conditions, we replaced peptone with a defined mix of amino acids based on the composition of peptone, an undefined digest of soy protein (Supplemental Information). When subcultured from the rich lysogeny broth (LB) broth, the *pabA* and *pabB* mutants grow well on this defined medium. However, after several generations of subculture in the defined medium, the *pabA* mutant was unable to grow without the addition of PABA. Growth was restored by as little as 50 nM PABA, whereas 100 μM folinic acid only incompletely rescued growth (Figure S4A).

We tested how modulating *E. coli* folate synthesis influenced *C. elegans* lifespan by maintaining worms on the *pabA* mutant grown on defined medium supplemented with a range of PABA concentrations. Before seeding, the *pabA* mutant was cultured in liquid defined medium containing 0.2 μM PABA. Subsequent growth of the *pabA* and wild-type bacterial lawn was the same at all PABA concentrations (Figure S4B). However, on medium supplemented with 0.1 μM PABA, *C. elegans* on the *pabA* mutant lived 39% longer than worms on wild-type bacteria (p = < 0.0001), or 50% longer than worms on *pabA* bacteria supplemented with 1 μM PABA (p = < 0.0001; Figure 4C). Supplementation of *pabA E. coli* with 0.2 μM PABA gave an intermediate result (28% increase, p = 0.0009, compared to 1 μM PABA). Increasing the concentration to 100 μM PABA did not decrease lifespan (Figure 4C) on either *pabA* or the wild-type bacteria, suggesting that PABA is not toxic. Rather, *C. elegans* lifespan is increased when excess folate synthesis is removed.

## Discussion

### *C. elegans* Folate and *C. elegans* Lifespan

Inhibiting *E. coli* folate synthesis decreases *C. elegans* folates (Virk et al., 2012), but we have shown that these changes in *C. elegans* folate are not responsible for the increased lifespan. Lifespan is also unaffected by *C. elegans* folate cycle inhibition, by MTX (Figure 2C), or by supplementation of vitamin B12, which is required for a key step in the folate cycle (Watson et al., 2014).

### Intestinal Accumulation of *E. coli*: a Cause or Consequence of *C. elegans* Aging?

Our analysis of accumulation of *E. coli* in the intestines of individual animals (Figure 3) does not exclude accumulation as the mechanism by which folate synthesis influences lifespan, but our data are also consistent with intestinal accumulation of *E. coli* being a consequence, rather than a cause, of intestinal aging. The intestine loses its structure early in aging worms (Herndon et al., 2002, McGee et al., 2011) and an age-related delay in the passage of *E. coli* caused by decreased pharyngeal pumping, decreased defecation, or breakdown in lumenal structure, would likely lead to bacterial accumulation. To our knowledge, it has never been demonstrated that preventing bacterial accumulation increases *C. elegans* lifespan. Electron microscopy studies have failed to find evidence of *E. coli* OP50 invasion in the *C. elegans* intestine (McGee et al., 2011) (David Hall, personal communication) and strains of *E. coli* used in *C. elegans* experiments lack the O-antigen needed for invasive pathogenesis (Browning et al., 2013). Through increased contact with intestinal cells, accumulation in the intestine may enhance other mechanisms by which *E. coli* are pathogenic, thus accelerating a cycle of functional loss in the aging intestine.

Second, we uncoupled the effects of *E. coli* growth from the *E. coli* activity that accelerates aging in *C. elegans*. Though SMX does not slow *E. coli* growth, we suggest it prevents a process that is also prevented by kanamycin. This process might be a factor that is induced by high folate levels and requires kanamycin-sensitive translation. The *E. coli* screen showed that lifespan is increased by only a few mutations, while many mutations slow *E. coli* growth without extending *C. elegans* lifespan. Thus, an alternative to the growth-dependent model is that a specific *E. coli* activity shortens lifespan, and this activity is blocked by treatment with kanamycin and other antibiotics.

### How *E. coli* Influences *C. elegans* Aging

The comparison with kanamycin suggests that inhibiting folate synthesis removes a pro-aging activity rather than producing an anti-aging activity. A common factor of *E. coli* genes isolated in the screen such as *pabA, pabB, rpoS, tatC, znuB*, and *ompA,* is that they reduce virulence when mutated in a wide range of pathogenic bacteria (Brown and Stocker, 1987, Dong and Schellhorn, 2010, Gabbianelli et al., 2011, Ochsner et al., 2002, Teng et al., 2006). Lab strains of *E. coli* used for *C. elegans* culture do not have known virulence factors, but the genes isolated in the screen might regulate other, as yet unknown, factors with a milder, long-term effect on their hosts. The decreased aversion to *E. coli* mutants identified in the screen, or to *E. coli* treated with SMX, is consistent with the removal of a toxin (Figure S3C) (Melo and Ruvkun, 2012). It is likely that some of the many peptides and compounds secreted by *E. coli* are toxic to *C. elegans*. These products may or may not influence lifespan. This study suggests that *E. coli* shorten *C. elegans* lifespan through a form of toxin-based virulence that is milder than observed with human pathogens, but may nevertheless be important for chronic disease and aging.

It is interesting to note that many *E. coli* genes and processes were not found to increase *C. elegans* lifespan robustly in the screen. Surprisingly, genes involved in ubiquinone synthesis or respiration were not found (Saiki et al., 2008), although the Keio *ubiG* mutant did not grow sufficiently to be included in the screen. The *E. coli* genes of unknown function, which constitute over a third of the mutant collection (Baba et al., 2006), were not screened and may be important for interactions with the host by, for example, synthesizing as yet uncharacterized toxins.

### The Role of Bacterial Folate

Our work suggests that in bacteria, folate has functions beyond its role in biosynthetic one-carbon metabolism. Mutation of *pabA* or *pabB* attenuates virulence in invasive bacteria, and the accepted explanation is that these mutants cannot grow in mammalian cells that lack PABA (Brown and Stocker, 1987, Chimalapati et al., 2011). However, another explanation is that these mutants are less able to produce toxins. Likewise, sulfonamides, which are less effective than most antibiotics in stopping bacterial growth, may be effective by preventing toxicity rather than growth. RpoS is an *E. coli* sigma factor activated in stationary phase to coordinate a global stress response, which includes increased virulence. Of the hundreds of genes reported to be under RpoS transcriptional control, *pabA* in *E. coli* (Weber et al., 2005) and *pabB* in *Bacillus subtilis* (Eymann et al., 2002) have been implicated in microarray experiments. Thus, folate synthesis may be stimulated by RpoS activity.

### Possible Implications for Human Aging and Disease

We have presented evidence that *E. coli* accelerates *C. elegans* aging independently of *E. coli* growth and *C. elegans* folate metabolism. If a similar relationship existed in the human gut, molecular characterization of this mechanism may uncover targets to intervene in aging and chronic disease. Chronic conditions such as obesity and inflammatory bowel disease are characterized by a dysbiosis of the microbiota, leading to an overgrowth of gamma proteobacteria, such as *E. coli*. (Winter and Bäumler, 2014). Interestingly, small intestine bacterial overgrowth leads to increased levels of serum folate, originating from dominating opportunistic bacteria (Camilo et al., 1996, Lakhani et al., 2008). Dysbiosis and the consequent overabundance of gamma proteobacteria may be an important factor in aging (Clark et al., 2015). This study shows that bacteria folate synthesis can be targeted without compromising host folate status, which can be maintained by selective supplementation.

## Experimental Procedures

### *C. elegans* Strains

N2 (wild-type), SS104 *glp-4(bn2)*, UF208 (wild-type), and UF209 *gcp-2.1(ok1004)*. See Supplemental Information for *gcp-2.1* analysis.

### *E. coli* Strains

OP50 *ura* (Brenner, 1974), OP50-GFP (containing plasmid pFPV25.1) (Labrousse et al., 2000), BW21153 (Keio collection wild-type), and WT Kan: BW21153 pGreen 0029 (Kan+). Strains from Keio collection as listed (Table S2). Keio strains transformed with complementation plasmids (Tables S1 and S3).

### Culture Conditions

NGM was prepared using high purity agar as described (Virk et al., 2012). As necessary, 25 μg/ml carbenicillin was added to maintain plasmid selection (OP50-GFP and complemented Keio mutants). Kanamycin was added after 24 hr of bacterial growth as described (Garigan et al., 2002). Folinic acid, folic acid, and MTX were from Schircks Laboratories. For the defined media, peptone was replaced by purified amino acids (Supplemental Information) and 0.2× trace metals (Studier, 2005). Calcium chloride was omitted as it is in the trace metals.

### Lifespan Analysis

Survival analyses were performed as described (Virk et al., 2012). Worms were cultured at 15°C and shifted to 25°C at the L3 stage. At 25°C, *E. coli* metabolism is more active than at lower temperatures (Virk et al., 2012). At the L4/young adult stage, animals were placed on bacteria under the experimental conditions. All lifespan data is in Table S1. Statistical significance was determined using log rank and Wilcoxon tests of the Kaplan-Meier survival model.

### GFP Bacterial Accumulation Assay

Worms were prepared and set up as for lifespan analysis using OP50-GFP. From day 5, using a Leica M165 FL stereomicroscope with a GFP2 filter with a 510 nm + long pass emission spectrum, individual animals were scored every 2 days for survival and as having no accumulation, partial accumulation, or full accumulation (Figure S1).

### Motility Assay

Animals were prepared as for lifespan analysis as described, with 16 plates per condition used and ten worms per plate. Animals were classified as described (Herndon et al., 2002).

### *C. elegans* Growth/Body Size Analysis

Animals were imaged after 2 days of 25°C growth after a synchronized egg lay. Images were analyzed for body length as described in Supplemental Information.

### Screening Method

See Supplemental Information.

### Gene Complementation of Keio Mutants

The relevant genomic region was PCR amplified from BW21153 using primers with restriction sites (Table S3), cloned into the low copy pMMB67EH Amp^R^ plasmid (Virk et al., 2012), and transformed into the relevant mutant strain. The plasmid was used as a control.

### Measuring *E. coli* Lawn Growth

To quantify bacteria in bacterial lawns, 2 ml of M9 was added to a 6 cm plate. The lawn was scraped off with a glass scraper. The liquid was then removed to a fresh tube. The total volume of the removed liquid was multiplied by the OD_600_ after a 5-fold dilution to generate a relative measure of bacteria.

### Aversion Assay

Aversion was measured as number of worms off the lawn/total number of worms as described in Melo and Ruvkun (2012).

## Author Contributions

Conceptualization; Supervision; and Funding Acquisition, D.W.; Methodology, D.W., B.V., J.J., C.A.M., A.R., N.C., and N.H.; Investigation, B.V., J.J., C.A.M., A.R., J.L., Y.L., M.C., and N.C.; Formal Analysis, B.V., C.A.M., J.J., D.W., and S.A.R.; and Writing, D.W., C.A.M., B.V., J.J., and S.A.R.

## Supplemental Information

Document S1. Supplemental Experimental Procedures and Figures S1–S4

Table S1. Lifespan Summaries, Related to Figures 2 and 4 The table contains conditions, numbers, and relevant statistical analysis for all lifespan experiments in the study.

Table S2. Keio Collection Strains Included in the Screen, Related to Figure 4 The table shows (A) strains tested and their survival in the first round. The table also shows (B) strains excluded because they did not grow on NGM plates

Table S3. Strains Selected for Second, Third, and Fourth Rounds of the Screen and the Results, Related to Figure 4 The table includes primers used to make complementation plasmids and the corresponding strains. The strains in red were added to second and third rounds because they were of interest.

Document S2. Article plus Supplemental Information
