# Supplementary material for: Folate Acts in E. coli to Accelerate C. elegans Aging Independently of Bacterial Biosynthesis
Source: Cell Rep. 2016 Feb 11;14(7):1611–20. doi: 10.1016/j.celrep.2016.01.051 (PMC4767678; doi:10.1016/j.celrep.2016.01.051)
Supplement: Document S1. Supplemental Experimental Procedures and Figures S1–S4 [file mmc1.pdf]

Cell Reports, Volume *14*

Supplemental Information

Folate Acts in *E. coli* to Accelerate *C. elegans* Aging Independently of Bacterial Biosynthesis

Bhupinder Virk, Jie Jia, Claire A. Maynard, Adelaide Raimundo, Jolien Lefebvre, Shane A. Richards, Natalia Chetina, Yen Liang, Noel Helliwell, Marta Cipinska, and David Weinkove

Supplemental Information

Figure S1

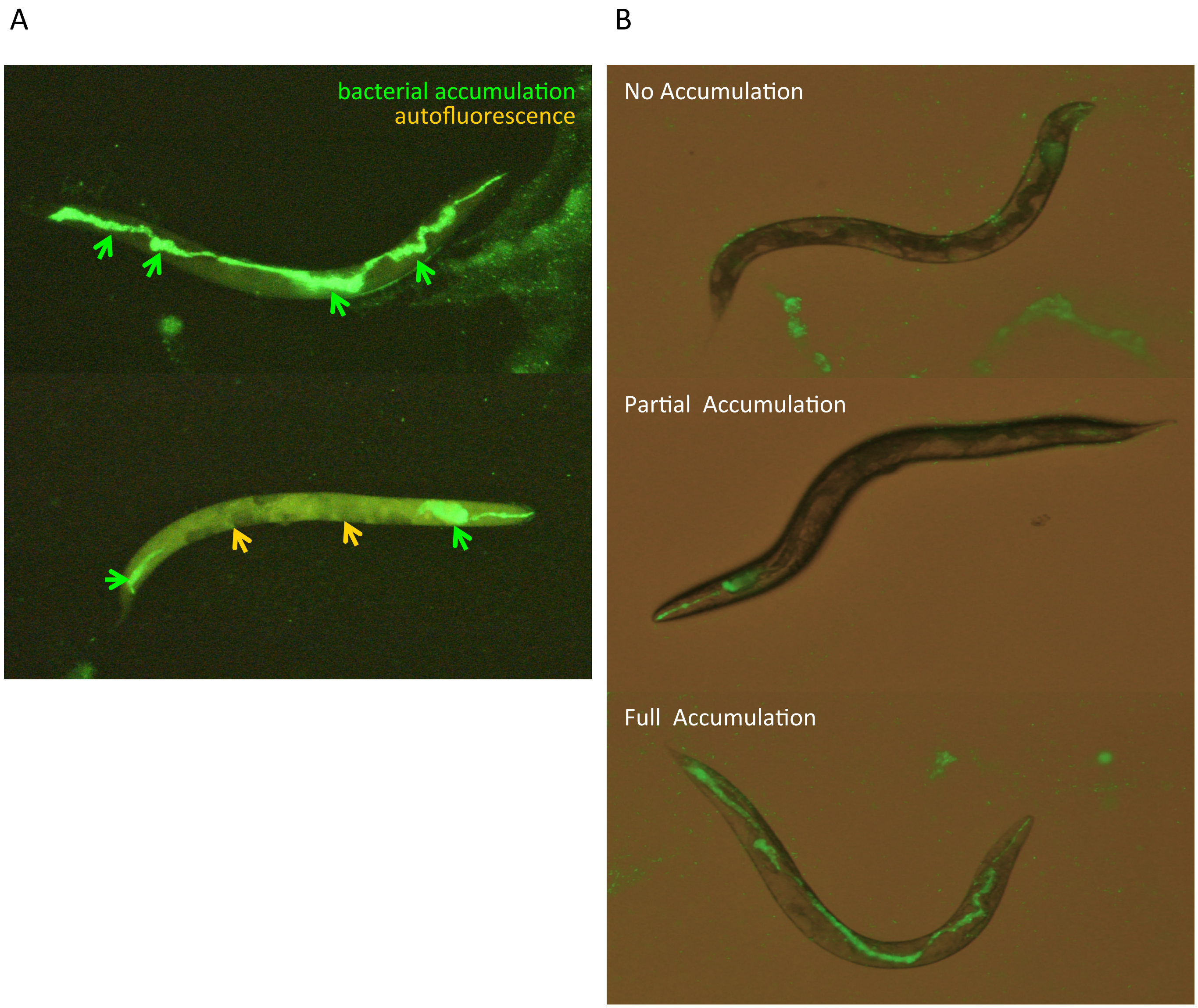

Figure S1. Distinguishing intestinal accumulation of *E. coli* OP50-GFP. Related to Figure 3. A) Representative images of single worms (observed using a long-pass green filter) illustrating the distinction between gut auto-fluorescence (yellow-green, yellow arrows) and GFP-expressing *E. coli* (bright green, green arrows). B) Representative images of single worms as classified according to extent of colonization by GFP-expressing *E. coli.*

Figure S2

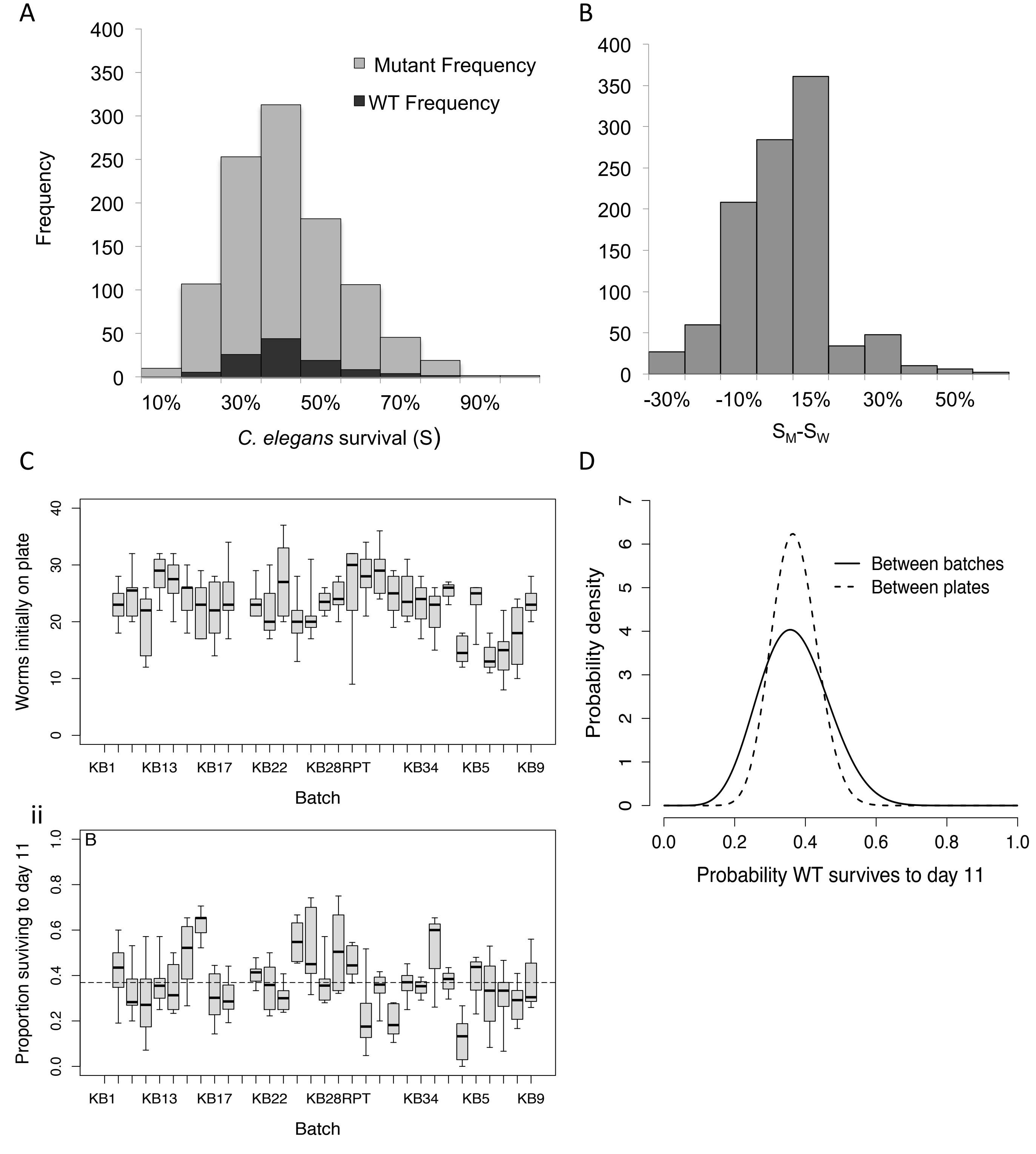

Figure S2. Estimating batch and plate variation on worm survival. Related to Figure 4. A) Histogram comparing the distribution in *C. elegans* survival in mutant and wild type strains. B) Normalizing the distribution to account for batch-to-batch variation: mutant survival (S_M) – wild type survival in the same batch (S_W). C) i) Variation in the number of WT worms scored per plate across batches ii) and the proportion of worms that survived in each plate. The dashed line indicates the maximum-likelihood fit for the overall survival fraction, *p*. All whiskers indicate the most extreme values. D) Best fitting beta distributions describing the variation in mean WT survival fraction to day 11 among plates (solid line), and variation in mean survival fraction between plates within a batch (dashed line). Here, we have depicted among plate variation for a batch having the overall mean survival, *p*. See Supplemental Experimental Procedures.

Supplemental Tables

(Excel Files)

Table S1. Lifespan summaries. **Related to Figures 2 and 4**. Contains conditions, numbers and relevant statistical analysis for all lifespan experiments in the study.

Table S2 Keio collection strains included in the screen. **Related to Figure 4**. A) Strains tested and their survival in the first round. B) Strains excluded because they did not grow on NGM plates.

Table S3 Strains selected for 2nd, 3rd and 4th rounds of the screen and the results. **Related to Figure 4.** Includes primers used to make complementation plasmids and the corresponding strains. Strains in red were added to 2nd and 3rd rounds because they were of interest.

Figure S3

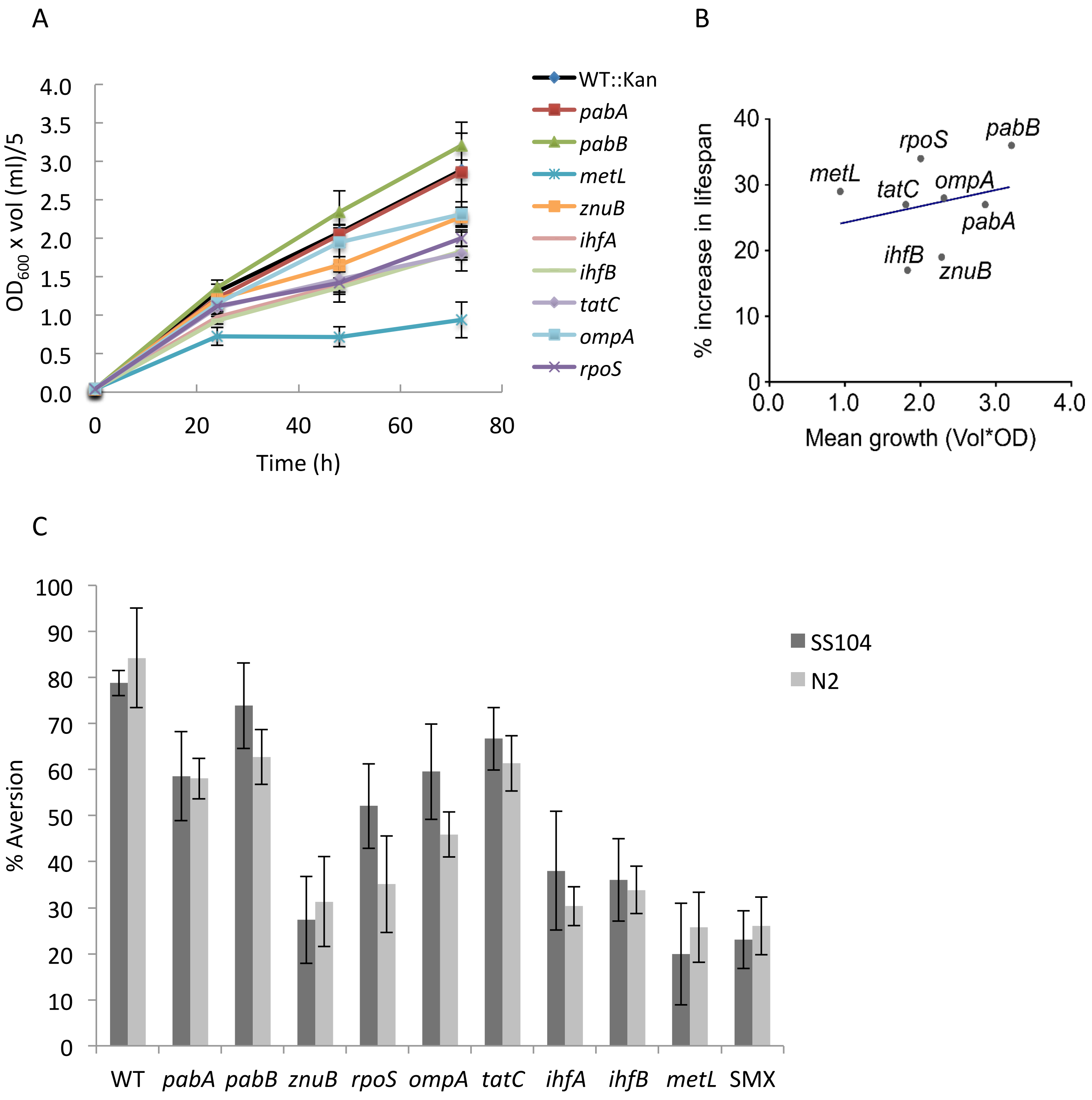

Figure S3. **Analysis of the 9 mutants isolated in the screen. Related to Figure 4.** A) Growth on NGM plates over 72 hours is significantly lower than wild type apart from *pabA* (no statistical difference), and *pabB* (significantly higher). See Experimental Procedures. Error bars represent standard deviation. B) Positive but not statistically significant correlation between bacterial growth on plates (A) and *C. elegans* lifespan increase for positive mutants from screen (Table S3). $R^2 = 0.06$, $P = 0.53$. C) Aversion of *C. elegans* strains to the bacterial lawn is decreased with the isolated mutants or with SMX. Error bars represent standard deviation. Aversion was statistically lower than control ($P < 0.01$) apart from on deletions of *pabB*, *ompA*, *tatC*, for which aversion is statistically lower only when data for N2 and SS104 are combined.

Figure S4

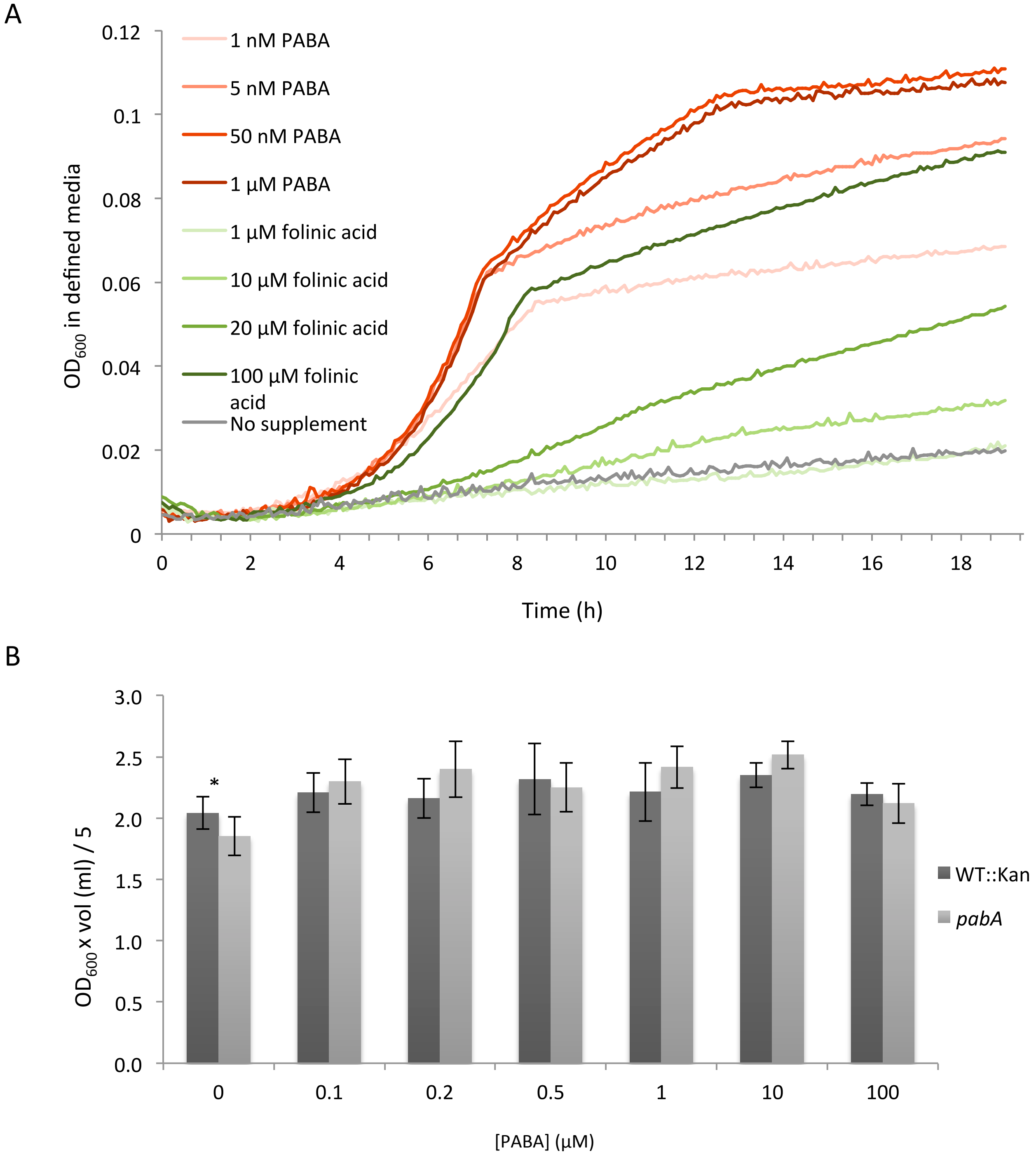

Figure S4. Growth of the *pabA* mutant. Related to Figure 4C and Figure 1. A) Growth curve of the *pabA* mutant in liquid defined media (Experimental Procedures) shows that 50 nM PABA is sufficient to restore bacterial growth. Folinic acid cannot completely restore growth at even at 100 µM. B) Growth on defined media plates with various concentrations of PABA after 72 hours, comparing *pabA* with WT under conditions used for Figure 4C. See Experimental Procedures. Error bars represent standard deviation. * p = < 0.01 for growth on *pabA* being less than growth on WT Kan.

Supplemental Experimental Procedures

GCP-2.1 characterization and analysis. Related to Figure 1

Three predicted proteins in *C. elegans* are encoded by R57.1 (named *gcp-2.1*), C35C5.2 (named *gcp-2.2*) and C35C5.11 (named *gcp-2.3*). All show similarity to mammalian GCPII amino acid sequences. The *gcp-2.1*(*ok1004*) deletion mutant from the *C. elegans* knockout consortium was outcrossed 3 times using N2 to make UF209 *gcp-2.1(ok1004).* The wild type sibling strain UF208 was used as the control. To make the *gcp-2.1* genomic transgene, a genomic fragment containing the predicted *gcp-2.1* gene was amplified using the primers R57gen_5: CTTAGGTTGGATCTCGTTGCTTGC and R57gen_3: TGTGTGGAAAGTGTGGTGAAGC using N2 genomic DNA as a template. 10 ng/µl of the PCR fragment with 90 ng/µl of marker plasmid *gpb-2*::*GFP* (van der Linden et al., 2001) was injected into UF209 *gcp-2.1(ok1004)* worms. A line transmitting the transgene mosaically, as assessed by GFP expression, was isolated (UF215 *gcp-2.1(ok1004) gqEx37[gcp-2.1, Pgbp-2::GFP].*). The transgene rescued the phenotypes of the *gcp-2.1* mutant.

Image analysis for worm growth. Related to Figure 1

Plates were imaged at 4.0x magnification using a Leica M165 FL stereomicroscope. The images were opened in ImageJ (Schneider et al., 2012) and the zoom function applied so that each image was 150% its original size, to enable more accurate measurement. The freehand line tool was then used to trace along the side of the body of each worm and the resulting line measured. To minimize bias, animals were selected for measurement randomly, using a grid overlay and then a random number generator to specify a grid reference. All animals in this specified square were then measured until a total of 30 worms had been measured for each condition. If an animal occupied two or more squares it was not measured.

Screening method. Related to Figure 4

Temperature sensitive sterile *glp-4(bn2)* worms were used in the screen. They were maintained at 15°C on OP50 and partly synchronized with an overnight timed egg lay. Three days later they were shifted to 25°C and then on the following day L4 worms were transferred to plates containing the bacterial strains to be

screened (Table S2A). Strains reported by Baba *et al.* to grow poorly in LB (<0.4 OD after 22 hours) and 15 strains that grew noticeably poorly on NGM plates were excluded (Table S2B). The screen plates were seeded 48 hours beforehand and left to grow at room temperature. The plates contained kanamycin (20 μg/ml) to kill the OP50. OP50-GFP was used to monitor bacterial persistence. For each strain at least 25 worms per plate were placed on at least 3 plates. For each batch, which consisted of 30-80 strains, 9 plates of 25+ worms were set up on a wild type BW21153 transformed with the Kan-containing plasmid pGreen 0029 (WT Kan). Plates were maintained at 25°C and survival was scored at either 11 or 12 days after the first day of adulthood. WT Kan plates were scored at both day 11 and 12. Survival was scored as number of alive worms/total number of worms. Strains that showed at least 15% increased survival (S_M) compared to the wild type control (S_W) were tested a second round of the screen (Table S3). Strains that passed this round, along with three other strains of interest (Table S3), were verified by PCR and tested in a full lifespan analysis using over 130 worms per strain in which survival was scored every two or three days. (Table S3). For strains that increased lifespan significantly in this third round, we complemented the missing gene with a wild type copy carried on a low copy plasmid (Table S3). The mutant *ihfA*, due to difficulties cloning the complementation construct, was confirmed by testing the "even" strain from the Keio collection (Table S1).

Estimating batch and plate variation on worm survival. Related to Figure 4A and Figure S2

First, we quantified the degree of variation in wild type (WT) survival both between batches and between plates within a batch. For simplicity we restricted our analysis to worms that were scored on day 11 (we find similar results when we examine worms scored on day 12). Let B be the number of batches performed and let P_i be the number of WT plates scored on day 11 in batch i. In total, we estimated variation in WT survival by day 11 from 209 plates distributed across B = 30 batches. The number of worms initially on a plate varied from 8 to 37 (mean = 22.66 worms), and the number of plates per batch varied from 3 to 12 (mean = 6.97 plates) (Figure S2Ci). Suppose the variation in the mean WT survival among batches has a beta-distribution with mean $\bar{p}$ and variance $\bar{p}(1-\bar{p})\emptyset_b/(1+\emptyset_b)$ (see (Richards, 2008) for details). Also, suppose that variation in mean survival among plates within a batch also has a beta-distribution with variance parameter $\emptyset_p$. These assumptions seem reasonable when inspecting the raw data describing the

between batch and between plate variation in survival fractions (Figure S2Cii). Under these assumptions, if n_{ij} of the N_{ij} worms on plate j in batch i survive to day 11, then the likelihood of the model, described by the set of parameters: $\boldsymbol{q} = \{\bar{p}, \emptyset_\mathrm{b}, \emptyset_\mathrm{p}\}$, is given by

$$L(\boldsymbol{\theta}|\mathrm{data}) = \prod_{i=1}^{B} \int_{p=0}^{1} f_\mathrm{b}(p|\bar{p}, \emptyset_\mathrm{b}) \prod_{j=1}^{P_i} f_\mathrm{bb}\left(n_{ij}|N_{ij}, p, \emptyset_\mathrm{p}\right) dp, \qquad (1)$$

where f_b and f_bb are the probability density functions for the beta and the beta-binomial distributions (see (Richards, 2008) for details). Likelihood ratio tests (LRTs) were used to evaluate if $\emptyset_\mathrm{b}$ or $\emptyset_\mathrm{p}$ were significantly different from zero (i.e. to test if there is no between batch or between plate variation). We found strong evidence of variation in WT survival between plates (LRT, $G_1 = 52.6$, $P < 0.001$) and variation in survival between plates within a batch (LRT, $G_1 = 11.7$, $P = 0.001$). Our best-fitting model was described by $\boldsymbol{q} = \{\bar{p} = 0.369, \emptyset_\mathrm{b} = 0.042, \emptyset_\mathrm{p} = 0.017\}$, which, as expected, predicts between batch variation being greater than between plate variation (Figure S2D).

Defined medium amino acid mix

The amino acid mix was designed using the composition of soy peptone. Together with trace metals, the mix was used to replace peptone in NGM (See Experimental Procedures for details). The concentration of the total mix was adjusted downwards so that growth of *E. coli* on defined medium agar plates was similar to that on peptone agar plates. Final concentrations (g/L): Alanine (1.419), Arginine (1.293), Aspartic acid (1.782), Cysteine (0.102), Glutamic acid (2.994), Glycine (3.414), Histidine (0.282), Isoleucine (0.468), Leucine (0.846), Lysine (0.924), Methionine (0.219), Phenylalanine (0.549), Proline (2.064), Serine (0.843), Threonine (0.534), Tryptophan (0.057), Tyrosine (0.318), Valine (0.627).

Supplemental References

Richards, S.A. (2008). Dealing with Overdispersed Count Data in Applied Ecology. Journal of Applied Ecology *45*, 218-227.

Schneider, C.A., Rasband, W.S., and Eliceiri, K.W. (2012). NIH Image to ImageJ: 25 years of image analysis. Nature Methods *9*, 671-675.

van der Linden, A.M., Simmer, F., Cuppen, E., and Plasterk, R.H. (2001). The G-protein beta-subunit GPB-2 in Caenorhabditis elegans regulates the G(o)alpha-G(q)alpha signaling network through interactions with the regulator of G-protein signaling proteins EGL-10 and EAT-16. Genetics *158*, 221-235.
