# Supplementary material for: Folate Acts in E. coli to Accelerate C. elegans Aging Independently of Bacterial Biosynthesis
Source: Cell Rep. 2016 Feb 11;14(7):1611–20. doi: 10.1016/j.celrep.2016.01.051 (PMC4767678; doi:10.1016/j.celrep.2016.01.051)
Supplement: Document S2. Article plus Supplemental Information [file mmc5.pdf]

Cell Reports

Report

Folate Acts in *E. coli* to Accelerate *C. elegans* Aging Independently of Bacterial Biosynthesis

Graphical Abstract

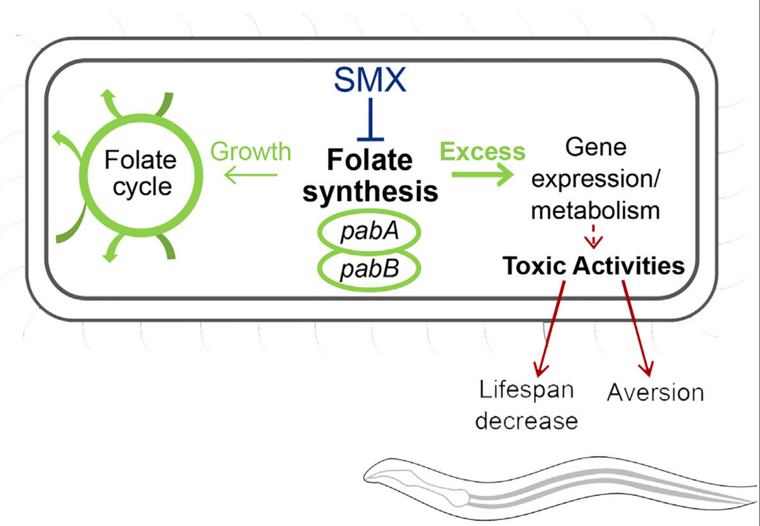

Highlights

- Limiting folate in *E. coli*, not in *C. elegans*, increases worm lifespan
- An *E. coli* screen for worm longevity identifies folate synthesis as a target
- Folate synthesis influences *E. coli* physiology independently of growth
- Bacterial folate synthesis may be a sustainable target for chronic disease

Authors

Bhupinder Virk, Jie Jia,
Claire A. Maynard, ..., Noel Helliwell,
Marta Cipinska, David Weinkove

Correspondence

david.weinkove@durham.ac.uk

In Brief

Virk et al. show that inhibiting *E. coli* folate synthesis does not increase *C. elegans* lifespan through changes to *C. elegans* folate, but by acting through an *E. coli* activity. They find nine *E. coli* mutants that extend lifespan. The gene identities suggest that mild bacterial toxicities accelerate host aging.

CrossMark
Virk et al., 2016, Cell Reports *14*, 1611–1620
February 23, 2016 ©2016 The Authors
http://dx.doi.org/10.1016/j.celrep.2016.01.051

CellPress

OPEN ACCESS
CellPress

Folate Acts in *E. coli* to Accelerate *C. elegans* Aging Independently of Bacterial Biosynthesis

Bhupinder Virk,[1,7,8] Jie Jia,[1,2,3,4,7] Claire A. Maynard,[1,7] Adelaide Raimundo,[1] Jolien Lefebvre,[1,5] Shane A. Richards,[1] Natalia Chetina,[1] Yen Liang,[1] Noel Helliwell,[1] Marta Cipinska,[1,6] and David Weinkove[1,6,*]
[1]School of Biological and Biomedical Sciences, Durham University, South Road, Durham DH1 3LE, UK
[2]Department of Nutrition, Shanghai Jiao Tong University School of Medicine, Shanghai 200025, China
[3]Key Laboratory of Pediatric Gastroenterology and Nutrition, Shanghai Institute for Pediatric Research, Shanghai 200092, China
[4]Department of Clinical Nutrition, Xin Hua Hospital affiliated to SJTU School of Medicine, Shanghai 200092, China
[5]Department HIVB, VIVES, Wilgenstraat 32, 8800 Roeselare, Belgium
[6]Biophysical Sciences Institute, Durham University, South Road, Durham DH1 3LE, UK
[7]Co-first author
[8]Present address: Bioinformatics Department, The Babraham Institute, Cambridge CB22 3AT, UK
*Correspondence: david.weinkove@durham.ac.uk
http://dx.doi.org/10.1016/j.celrep.2016.01.051
This is an open access article under the CC BY license (http://creativecommons.org/licenses/by/4.0/).

SUMMARY

Folates are cofactors for biosynthetic enzymes in all eukaryotic and prokaryotic cells. Animals cannot synthesize folate and must acquire it from their diet or microbiota. Previously, we showed that inhibiting *E. coli* folate synthesis increases *C. elegans* lifespan. Here, we show that restriction or supplementation of *C. elegans* folate does not influence lifespan. Thus, folate is required in *E. coli* to shorten worm lifespan. Bacterial proliferation in the intestine has been proposed as a mechanism for the life-shortening influence of *E. coli*. However, we found no correlation between *C. elegans* survival and bacterial growth in a screen of 1,000+ *E. coli* deletion mutants. Nine mutants increased worm lifespan robustly, suggesting specific gene regulation is required for the life-shortening activity of *E. coli*. Disrupting the biosynthetic folate cycle did not increase lifespan. Thus, folate acts through a growth-independent route in *E. coli* to accelerate animal aging.

INTRODUCTION

Both nutrition and the host-associated microbiota are thought to impact longevity (Heintz and Mair, 2014; Rizza et al., 2014). Diet influences the metabolism of gut microbes, which in turn can synthesize nutrients for the host. These interactions make it difficult to unravel the contributions of diet and the gut microbiota to long-term health (Lozupone et al., 2012). This complexity can be addressed with model systems such as the nematode *Caenorhabditis elegans* (Collins et al., 2008). Yet even here, there are numerous interactions between the nutrient agar medium, the *Escherichia coli* bacterial lawn, and the worm. Chemical manipulations of the medium and genetic manipulations of both *E. coli* and *C. elegans* provide tools to understand these interactions (Weinkove, 2015).

Folates in their reduced tetrahydrofolate (THF) form are required as enzymatic cofactors in the folate cycle; a series of metabolic steps found in all cells (including both bacteria and animals) required for cell biosynthesis. Products include purines, pyrimidines, glycine, and methionine, which are required to generate the methyl donor molecule S-adenosyl methionine (SAM) (Bailey and Gregory, 1999). Animals cannot synthesize folates and so obtain folates from their diets and associated microbes (Asrar and O'Connor, 2005; Lakoff et al., 2014). Our previous research showed that *C. elegans* lifespan is increased when *E. coli* folate synthesis is disrupted either by a mutation in the gene *aroD*, which is needed to make aromatic compounds including the folate precursor para-aminobenzoic acid (PABA), or by sulfamethoxazole (SMX), a sulfonamide drug that competes with PABA for the active site of the enzyme dihydropteroate synthase (Virk et al., 2012). This enzyme is a key step in folate biosynthesis and is absent from animals. *C. elegans* obtains folates from *E. coli* and thus several possible mechanisms might explain why *E. coli* folate synthesis affects *C. elegans* lifespan. Distinguishing the effects of folates in bacteria and folates in their animal hosts is important because folate supplementation is beneficial to human health and any intervention would need to maintain healthy levels of serum folate.

Dietary, or caloric, restriction has been shown to extend the lifespan of *C. elegans* (Greer and Brunet, 2009; Mair and Dillin, 2008). SMX does not slow *E. coli* growth and therefore has no effect on food availability. Furthermore, *C. elegans* grow and reproduce normally (Virk et al., 2012). Thus, a limitation of macronutrients is an unlikely explanation. Alternatively, inhibition of *E. coli* folate synthesis may influence *C. elegans* lifespan by limiting dietary folate and/or a specific change in folate-dependent nutrients (Lee et al., 2015). For example, restriction of methionine increases lifespan in rodents and influences lifespan in *Drosophila* (Grandison et al., 2009; Sanchez-Roman and Barja, 2013). Mutation of *C. elegans sams-1,* the gene encoding SAM synthase, extends lifespan (Hansen et al., 2005).

The diabetes drug metformin increases *C. elegans* lifespan in a manner dependent on the *E. coli* strain and changes in *C. elegans* folate and methionine metabolism are implicated in mediating the lifespan extension (Cabreiro et al., 2013).

Another possible explanation is that folate synthesis inhibition increases *C. elegans* lifespan by altering *E. coli* physiology. *E. coli* can accumulate in the intestine of older *C. elegans* adults and because treatment of *E. coli* with antibiotics or UV increases worm lifespan, this accumulation is widely thought to accelerate *C. elegans* aging (Garigan et al., 2002; Gems and Riddle, 2000; McGee et al., 2011). More subtly, changes in bacterial toxicity caused by changes in bacterial metabolism might influence *C. elegans* aging. The *E. coli ubiG* mutant, which cannot synthesis coenzyme Q/ubiquinone, increases worm lifespan by influencing bacterial respiration rather than dietary intake of Q (Saiki et al., 2008).

Here, we show that modulating folate uptake or the folate cycle in *C. elegans* does not affect lifespan, suggesting *E. coli* folate influences *C. elegans* lifespan by acting on *E. coli* physiology. Apart from the Q synthesis genes and *aroD* (Saiki et al., 2008; Virk et al., 2012), little is known about how *E. coli* genetics influences *C. elegans* lifespan. A genetic screen of over 1,000 *E. coli* mutants shows that bacterial growth does not correlate with *C. elegans* survival and only a few specific interventions increase *C. elegans* lifespan, including the mutation of genes involved in *E. coli* folate synthesis, but not in the *E. coli* folate cycle. In addition to its role in bacterial growth, we propose that folate acts to change *E. coli* physiology in a way that accelerates *C. elegans* aging.

RESULTS

Genetic Disruption of *C. elegans* Folate Uptake and Restoration by Supplementation

To distinguish the effects of *C. elegans* folate from *E. coli* folate, we targeted folate uptake in *C. elegans*. The reduced folate carrier FOLT-1 takes up THFs across the intestinal epithelial membrane (Balamurugan et al., 2007). The published *folt-1* deletion allele causes sterility, so we turned to another *C. elegans* mutant predicted to disrupt folate uptake. In mammals, glutamate carboxypeptidase II (GCPII) cleaves glutamates from polyglutamated THFs in the gut, to create monoglutamated folates that are preferentially imported by folate carriers and transporters (Halsted et al., 1998). The *E. coli* diet contains predominantly polyglutamated THFs (Kwon et al., 2008; Virk et al., 2012), suggesting that *C. elegans* requires GCPII activity. There are three *C. elegans* genes that encode a GCPII homolog (Supplemental Information). Animals lacking the GCPII gene *gcp-2.1*, *WB Gene*: WBGene00020082, appear healthy and develop normally on *E. coli* OP50, but develop slowly and are uncoordinated and sterile on OP50 treated with 128 μg/ml SMX (Figure 1A). This phenotype is rescued completely by supplementation with 10 μM formyl THF monoglutamate, a naturally occurring reduced folate also known as folinic acid or leucovorin (Figures 1A and 1B). Folinic acid can rescue the *gcp-2.1* phenotype at a 20-fold lower concentration than can folic acid, the oxidized folate used commonly in dietary supplements (Figure 1B). These results are consistent with the specificity of FOLT-1 for reduced folates (Balamurugan et al., 2007) and a role for GCP-2.1 in folate uptake (Figure 1C).

E. coli does not have folate uptake transporters, but can synthesize folate from the folate breakdown products PABA, which can freely diffuse through membranes, and PAB-Glutamate, which is taken up by an active transport system (Hussein et al., 1998). Thus, folic acid restores folate synthesis in an *aroD* mutant, most clearly at concentrations of 100 μM or more (Virk et al., 2012). In the presence of SMX, which competes with PABA for the enzyme dihydropteroate synthase, it is likely that more folate breakdown products would be required to restore folate synthesis. For example, PABA is required at a concentration of 250 μM to fully reverse the lifespan extension caused by 505 μM (128 μg/ml) SMX (Virk et al., 2012). It is unlikely that 10 μM folinic acid would provide enough breakdown products to restore *E. coli* folate synthesis in competition with 505 μM SMX. Thus, we conclude that folinic acid rescues *gcp-2.1* by directly supplementing *C. elegans* folate (Figure 1C).

Modulation of *C. elegans* Folate Status Does Not Influence Lifespan

Turning to effects on aging, we found that the *C. elegans gcp-2.1* mutant has a similar, if not slightly shorter lifespan, than wild-type controls ($p = 0.0227$; Figure 2A). Furthermore, the increased *C. elegans* lifespan caused by SMX was unaffected by supplementation with 10 μM folinic acid (Figure 2B). Methotrexate (MTX), a dihydrofolate reductase inhibitor specific to animals, inhibits the *C. elegans* folate cycle. 100 μg/ml MTX causes developmental defects in *nuc-1* mutants and is five times the concentration required to cause this phenotype (Mello et al., 1991; Virk et al., 2012). MTX did not affect the lifespan of *C. elegans* and also failed to influence the lifespan of worms on SMX-treated bacteria (Figure 2C). Together, these results suggest that the SMX-induced lifespan increase cannot be explained by decreased *C. elegans* folate uptake or impaired folate-dependent *C. elegans* metabolism.

SMX and Kanamycin Treatment Cause an Identical Increase in *C. elegans* Lifespan

Treating *E. coli* with ultraviolet irradiation or antibiotics increases *C. elegans* lifespan, suggesting that *E. coli* possess a life-shortening activity (Garigan et al., 2002; Gems and Riddle, 2000). We compared OP50 treated with SMX, which does not influence *E. coli* viability, with OP50 treated with kanamycin, a bacterial translation inhibitor. Addition of kanamycin to the bacterial lawn stops cells forming further colonies (Virk et al., 2012). Using large cohorts, worms maintained on kanamycin-treated bacteria showed an almost identical survival curve to those on bacteria treated with SMX (25% increase in mean lifespan compared to wild-type, $p = < 0.0001$), with a small further increase in lifespan (4%, $p = 0.0008$) when both drugs were combined (Figure 2D). While there are many possible explanations for this result and SMX and kanamycin have very different targets and effects on *E. coli* metabolism, the lack of a substantial additive effect suggests that both drugs might inhibit a shared downstream process that shortens *C. elegans* lifespan.

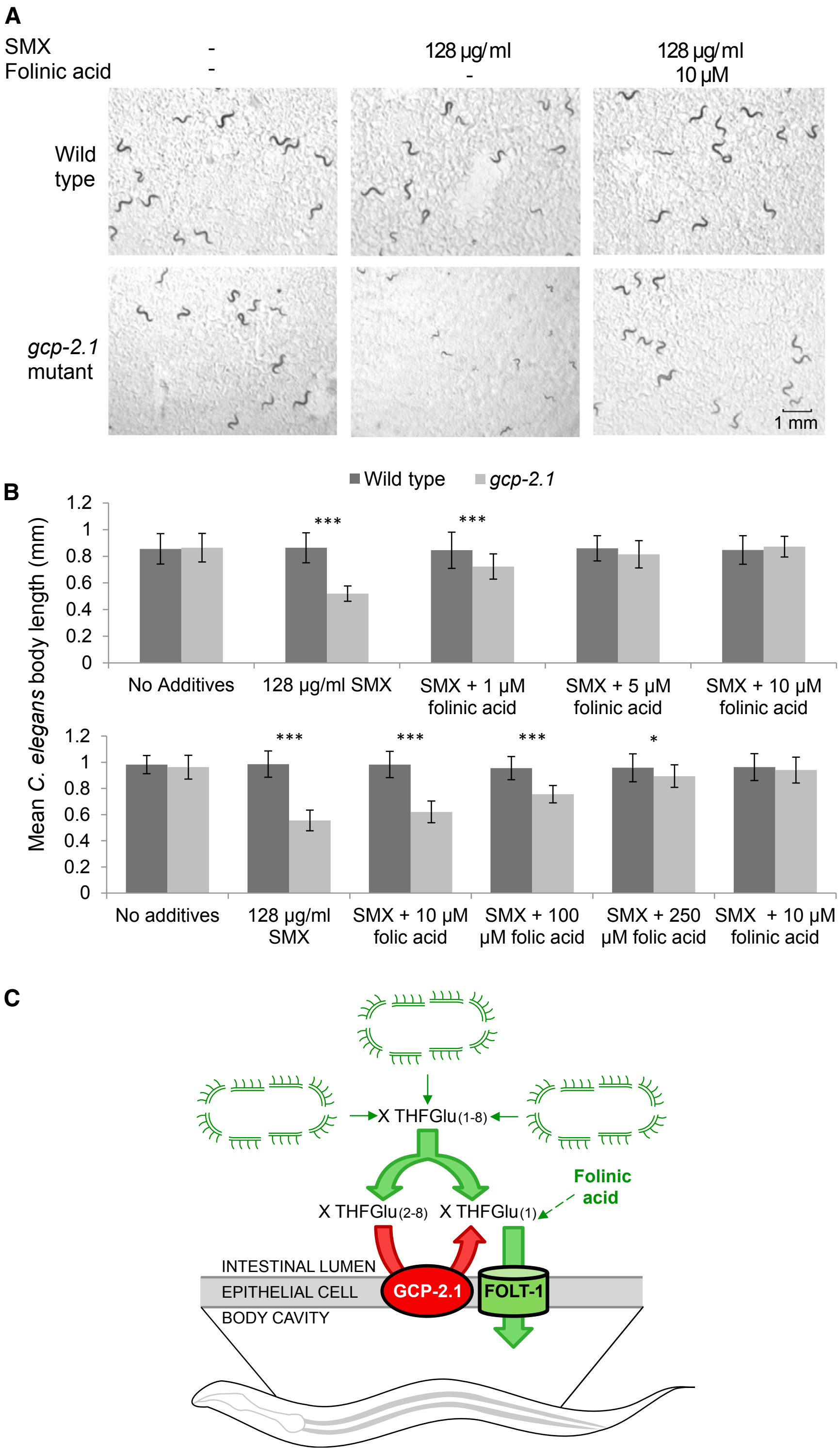

Figure 1. The *C. elegans gcp-2.1* Folate Uptake Mutant Is Sensitive to SMX-Treated *E. coli* and Is Rescued by Folinic Acid

(A) *gcp-2.1* mutants develop as wild-type worms on untreated OP50 *E. coli*, but SMX treatment delays the growth of *gcp-2.1* mutants, and this defect can be rescued by 10 µM folinic acid. The images were taken after 48 hr of growth at 25°C.

(B) Quantification of the growth, as measured by body length. The error bars represent SD. The *gcp-2.1* mutant growth on SMX is restored at 5 µM and 10 µM folinic acid and partially with 1 µM folinic acid (*$p < 0.05$, **$p < 0.01$, and ***$p < 0.005$) (t test). Folic acid can fully rescue growth only at 250 µM.

(C) Model showing that *E. coli* folate synthesis generates THFs with up to eight glutamate residues and various one carbon groups, $xTHFGlu_{1–8}$ (x = methyl, formyl, methenyl, and methylene). The *C. elegans* GCPII GCP-2.1 cleaves glutamate residues from $xTHFGlu_{2–8}$ to generate $xTHFGlu_1$, the preferred substrates of the *C. elegans* reduced folate transporter FOLT-1. Folinic acid, as an $xTHFGlu_1$ (5-formyl THF), can be taken up directly by FOLT-1, bypassing GCP-2.1.

It Is Unlikely that *E. coli* Shortens Lifespan Solely through Intestinal Accumulation

Kanamycin and similar treatments are thought to increase *C. elegans* lifespan by preventing *E. coli* accumulation in the intestinal lumen (Garigan et al., 2002). To test whether SMX prevents accumulation, we performed lifespan experiments with worms maintained on *E. coli* OP50 expressing GFP. These fluorescent bacteria could be observed accumulating in live worms from the beginning of adulthood through to death. A long pass green filter, which allows red light through, was used to distinguish GFP-expressing *E. coli* from gut autofluorescence (Figure S1). This distinction is harder to make using the narrow band green filters employed in most studies of GFP-expressing bacteria. We detected accumulation of bacteria in some worms from day 5 of adulthood onward. However, many worms did not appear to accumulate bacteria at all. Approximately 50% of recently dead worms showed no visible accumulation (Figure 3A), suggesting that bacterial accumulation did not contribute to their death. This heterogeneity in accumulation is missed when groups of worms rather than individuals are assessed (Gomez et al., 2012; Portal-Celhay et al., 2012). SMX decreased the number of worms that died with bacterial accumulation, but did not prevent accumulation from occurring in all animals. Across the whole lifespan, SMX delayed the onset of bacterial accumulation, but did not prevent it (Figure 3B). SMX causes the GFP expressing OP50 *E. coli* to appear brighter than in untreated conditions, confirming

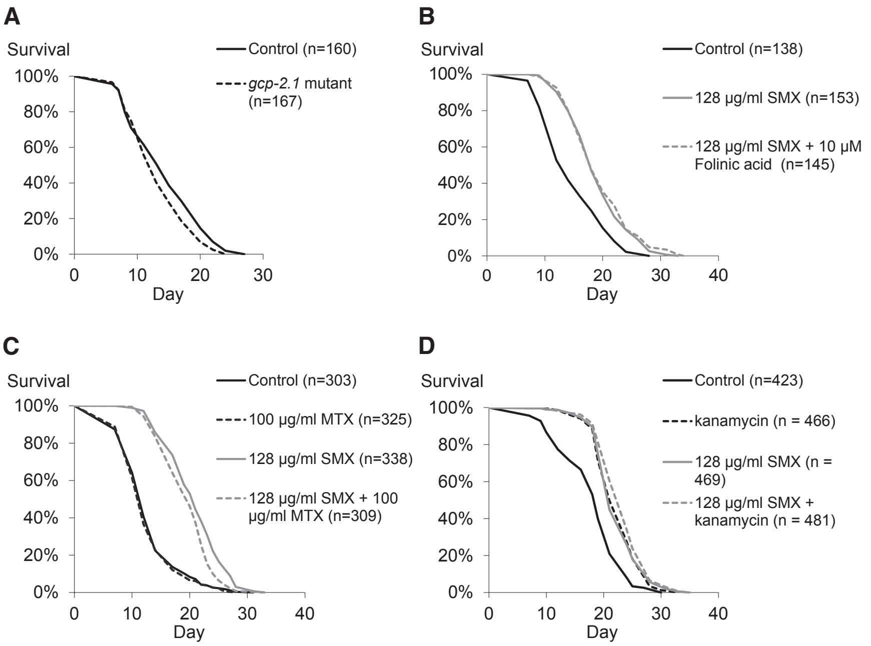

Figure 2. Lifespan Analyses of Perturbations to *C. elegans* Folate and Comparison of SMX and Kanamycin Treatment

(A) The *gcp-2.1(ok1004)* mutant does not increase lifespan.

(B) 10 μM folinic acid does not affect the lifespan extension caused by 128 μg/ml SMX.

(C) MTX has no effect on *C. elegans* lifespan.

(D) Kanamycin and SMX have a very similar effect on *C. elegans* lifespan. See Table S1 for lifespan conditions and statistics.

that these bacteria are capable of producing increased protein. SMX prolonged the time that *C. elegans* stay mobile, consistent with a decrease in the rate of aging (Figure 3C). The structure and function of the *C. elegans* intestine declines with age. Thus, like motility, bacteria accumulation is a biomarker, but not necessarily a cause, of aging.

E. coli Mutants Influence *C. elegans* Lifespan Independently of *E. coli* Growth

To further understand how *E. coli* influence *C. elegans* lifespan, we conducted a screen of over 1,000 *E. coli* K12 mutants from the Keio collection (Baba et al., 2006). We tested deletions in all non-essential genes predicted to encode enzymes in the folate cycle or related pathways (25 genes; Table S2) and deletions of 981 randomly selected genes of known function (Experimental Procedures; Table S2). Scoring across the lifespan is impractical for a large-scale screen, so we scored survival at a single time point (day 11/12) close to the median lifespan. This strategy allows greater statistical power than scoring near the end of the survival curve and the identification of strains that shorten, as well as extend, *C. elegans* lifespan.

The mutants were scored in batches. Each batch contained several mutants and three wild-type strains. The distribution in survival shown by these controls did not differ from the distribution across all mutants tested, suggesting that mutating single *E. coli* genes had no detectable large-scale effect on *C. elegans* lifespan (Supplemental Information; Figure S2). To account for batch-to-batch variation, we subtracted the mean survival of the wild-type in a batch, S_W, from the survival of each mutant S_M in that batch (Supplemental Information). S_M - S_W revealed a narrower distribution and a set of candidates for strains that increased *C. elegans* lifespan (Figure S2). Using growth data from Baba et al. (2006), we found no correlation between strain growth and survival of *C. elegans* (Figure 4A), suggesting that *E. coli* growth rate does not influence *C. elegans* aging.

Nine *E. coli* Mutants Robustly Increase *C. elegans* Lifespan

To be confident of identifying individual mutants that increased *C. elegans* lifespan, we repeated the screen for the 67 strains that caused worms to survive at least 15% more than on the control strain. We undertook full lifespan analysis of the 22 strains that passed this second round. Finally, we retested the 11 strains that passed this third round and included full lifespan analysis of the strains with the mutation complemented by the wild-type *E. coli* gene. This step ruled out lifespan increases from spontaneous second-site mutations (Supplemental Information). There were nine deletions that passed this final test, representing less than 1% of genes tested.

Three of the identified mutants (*metL*, *ihfA*, and *ihfB*) caused the bacterial lawn to appear more liquid than normal. This environment would have a strong influence on *C. elegans* physiology (Lewis and Fleming, 1995). The other mutants discovered had no visible effect on the *E. coli* lawn and so are more likely to influence lifespan through biological, rather than physio-chemical mechanisms. These mutants included a deletion of *rpoS*, a stationary phase sigma factor. This transcription factor regulates over 200 genes in response to low nutrients or other stresses (Battesti et al., 2011). Other deletions that extended lifespan included *tatC*, a gene encoding a component of the twin arginine translocation pathway, which transports folded proteins to the periplasm (Stanley et al., 2001), *ompA*, an abundant constituent of the outer membrane (Smith et al., 2007), and *znuB*, which encodes part of the ZnuABC zinc transporter, needed to take up zinc at low concentrations (Patzer and Hantke, 1998). Finally, we isolated deletions in *pabA* and *pabB* (Figure 4B). These genes encode two enzymes that associate and catalyze key steps in PABA synthesis (Green et al., 1996). In summary, the screen underlined the importance of *E. coli* folate synthesis in *C. elegans* lifespan regulation and identified other genes with diverse functions.

Apart from the *pabA* and *pabB* mutants, the mutants grow slower on the petri dish than the wild-type strain (Figure S3A). There was a positive, but not significant, correlation between extent of the lifespan extension and the growth rate of the strain

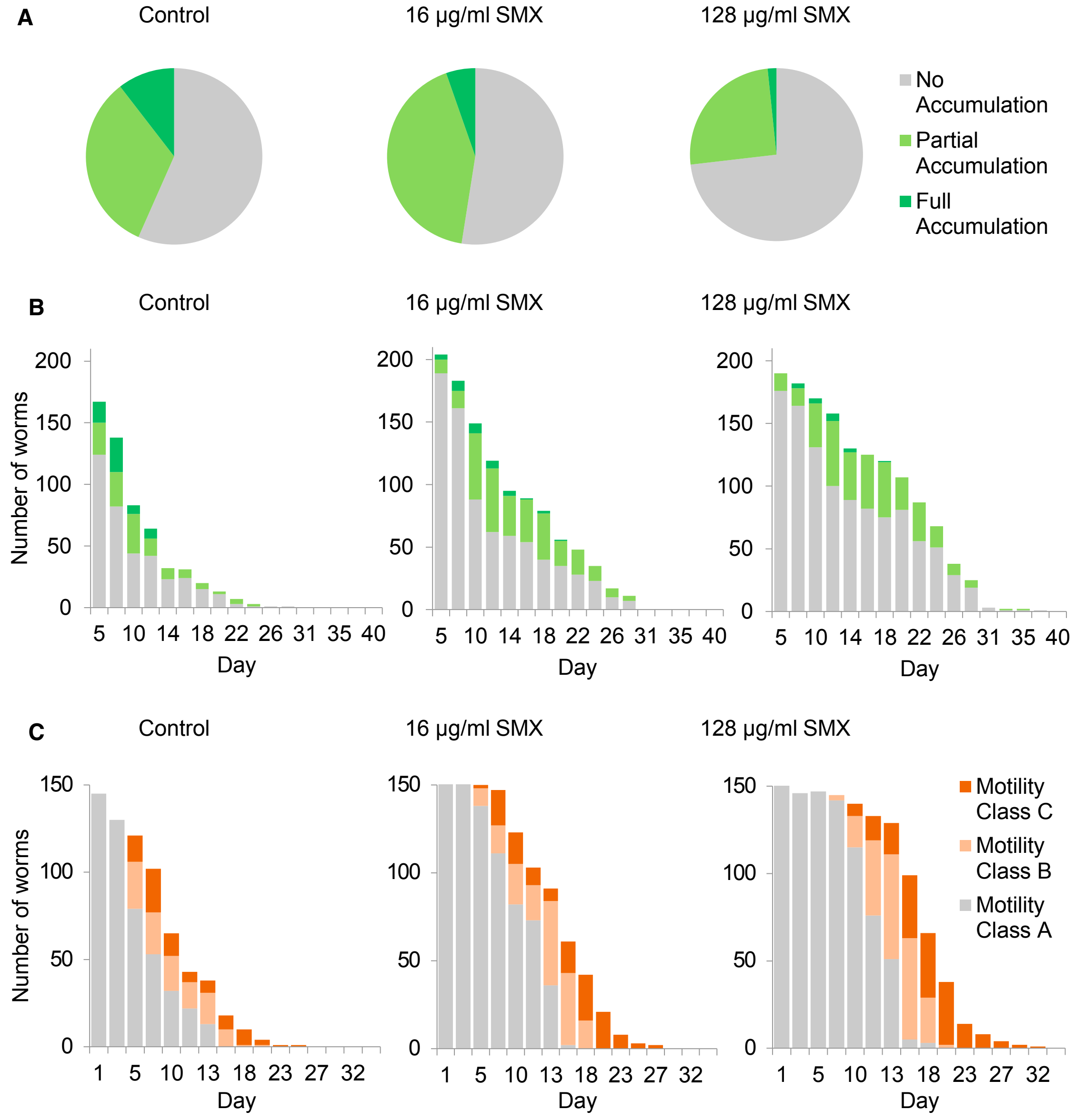

Figure 3. Intestinal Accumulation of Bacteria Does Not Occur in All Animals and Is Not Prevented by SMX

(A) Accumulation in recently dead animals as assessed by visualizing *E. coli* GFP in the intestinal lumen. The data are pooled from two biological replicates.

(B) Numbers of alive worms at indicated time points with classification of accumulation.

(C) Motility analysis of *glp-4(bn2)* worms on OP50 treated with 0, 16 µg/ml, and 128 µg/ml SMX. Each worm was scored as belonging to motility class A (constantly moving), B (moves when prodded), or C (twitches only) as described (Herndon et al., 2002).

(Figure S3B). We noticed that *C. elegans* spent more time on the bacterial lawn if the *E. coli* strain was one of the life-extending mutants or had been treated with SMX (Figure S3C). SMX treatment of OP50 also produced a similar decrease in aversion. Thus, the increased lifespan of *C. elegans* is not caused by decreased exposure to food or to *E. coli*. *C. elegans* avoids toxic bacteria, and this avoidance is thought to be triggered by perception of disruption to worm metabolism caused by bacterial toxins (Melo and Ruvkun, 2012). Thus, the mutations isolated in the screen, or chemical inhibition of folate synthesis, may remove toxicity from *E. coli.*

Disruption of the *E. coli* Folate Cycle Does Not Increase *C. elegans* Lifespan

Although the identification of *pabA* and *pabB* was consistent with *E. coli* folate synthesis inhibition increasing *C. elegans* lifespan, we were surprised that no genes involved in the folate cycle or related pathways were identified. An exception was *metL*, which encodes an enzyme needed in two early stages of methionine biosynthesis. However, deletions in other methionine synthesis genes did not have a lifespan phenotype. Because of the variation found in the screen, we wanted to make sure that we had not missed any folate-related genes.

OPEN
ACCESS
CellPress

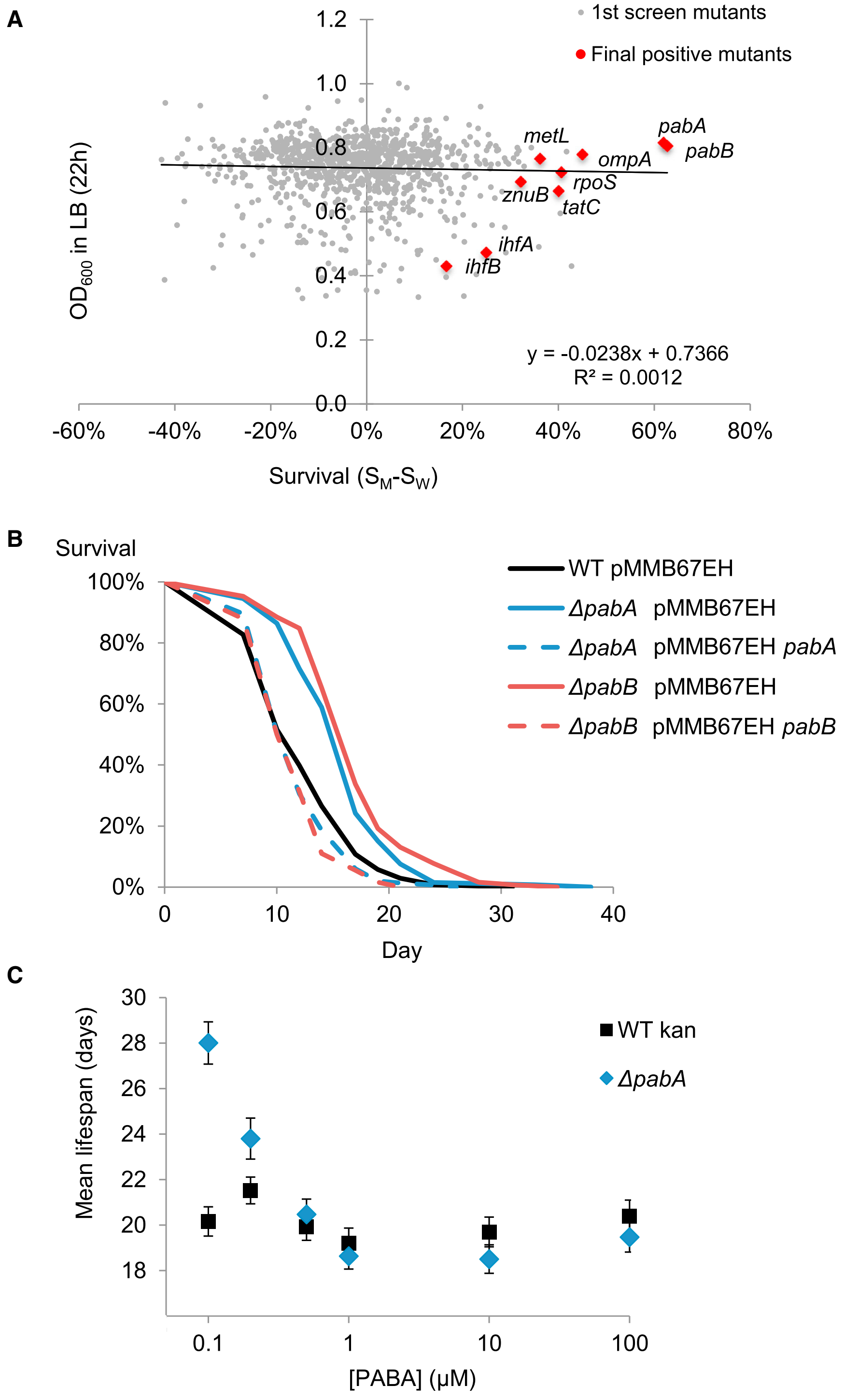

Figure 4. A Screen of *E. coli* Deletion Mutants for *C. elegans* Survival Identified Nine Mutants that Robustly Extend Lifespan

(A) No correlation between growth of mutant strains in LB (Baba et al., 2006) and normalized *C. elegans* survival at day 11 or 12. The mutants that increase lifespan after the fourth round of the screen are indicated.

(B) *pabA* and *pabB* mutants increase *C. elegans* lifespan, and this increase is reversed by gene complementation. See Table S1 for details.

(C) Mean lifespan (with SD) at various concentrations of PABA on defined media plates, comparing worms on the *E. coli pabA* mutant with worms on WT *E. coli*. At 0.1 and 0.2 μM PABA, the lifespans on *pabA* and wild-type *E. coli* are significantly different (see Results and Table S1 for details). The error bars represent SD.

We performed full lifespan analysis on *E. coli* mutants in 23 non-essential genes involved in the folate cycle or related metabolic pathways. None of these mutants extended lifespan apart from a small, but significant, effect of a deletion of *glyA* (Table S1). Thus *E. coli* folate synthesis, but not the folate cycle, which is needed for *E. coli* growth, limits *C. elegans* lifespan.

C. elegans Lifespan Can Be Altered through the Availability of PABA to *E. coli*

Folate synthesis is essential for *E. coli* growth because *E. coli* cannot take up intact folate. However, SMX or mutation of *pabA* or *pabB* do not decrease *E. coli* growth rate under the conditions that they increase *C. elegans* lifespan (Virk et al., 2012) (Figure S3A). Thus, these interventions must only remove folate in excess of that required for *E. coli* growth. To understand the relative levels at which folate synthesis is required to limit lifespan compared to requirements for growth, we took advantage of the fact that PABA synthesis in *E. coli* can be bypassed by adding exogenous PABA, which can diffuse across membranes and that *C. elegans* cannot make folate from PABA. The growth of *pabA* and *pabB* mutants on peptone-based NGM, suggests this medium contains a source of PABA. To remove this PABA while minimizing changes to the nutritional conditions, we replaced peptone with a defined mix of amino acids based on the composition of peptone, an undefined digest of soy protein (Supplemental Information). When subcultured from the rich lysogeny broth (LB) broth, the *pabA* and *pabB* mutants grow well on this defined medium. However, after several generations of subculture in the defined medium, the *pabA* mutant was unable to grow without the addition of PABA. Growth was restored by as little as 50 nM PABA, whereas 100 μM folinic acid only incompletely rescued growth (Figure S4A).

We tested how modulating *E. coli* folate synthesis influenced *C. elegans* lifespan by maintaining worms on the *pabA* mutant

grown on defined medium supplemented with a range of PABA concentrations. Before seeding, the *pabA* mutant was cultured in liquid defined medium containing 0.2 μM PABA. Subsequent growth of the *pabA* and wild-type bacterial lawn was the same at all PABA concentrations (Figure S4B). However, on medium supplemented with 0.1 μM PABA, *C. elegans* on the *pabA* mutant lived 39% longer than worms on wild-type bacteria (p = < 0.0001), or 50% longer than worms on *pabA* bacteria supplemented with 1 μM PABA (p = < 0.0001; Figure 4C). Supplementation of *pabA E. coli* with 0.2 μM PABA gave an intermediate result (28% increase, p = 0.0009, compared to 1 μM PABA). Increasing the concentration to 100 μM PABA did not decrease lifespan (Figure 4C) on either *pabA* or the wild-type bacteria, suggesting that PABA is not toxic. Rather, *C. elegans* lifespan is increased when excess folate synthesis is removed.

DISCUSSION

C. elegans Folate and *C. elegans* Lifespan

Inhibiting *E. coli* folate synthesis decreases *C. elegans* folates (Virk et al., 2012), but we have shown that these changes in *C. elegans* folate are not responsible for the increased lifespan. Lifespan is also unaffected by *C. elegans* folate cycle inhibition, by MTX (Figure 2C), or by supplementation of vitamin B12, which is required for a key step in the folate cycle (Watson et al., 2014).

Intestinal Accumulation of *E. coli*: a Cause or Consequence of *C. elegans* Aging?

Our analysis of accumulation of *E. coli* in the intestines of individual animals (Figure 3) does not exclude accumulation as the mechanism by which folate synthesis influences lifespan, but our data are also consistent with intestinal accumulation of *E. coli* being a consequence, rather than a cause, of intestinal aging. The intestine loses its structure early in aging worms (Herndon et al., 2002; McGee et al., 2011) and an age-related delay in the passage of *E. coli* caused by decreased pharyngeal pumping, decreased defecation, or breakdown in lumenal structure, would likely lead to bacterial accumulation. To our knowledge, it has never been demonstrated that preventing bacterial accumulation increases *C. elegans* lifespan. Electron microscopy studies have failed to find evidence of *E. coli* OP50 invasion in the *C. elegans* intestine (McGee et al., 2011) (David Hall, personal communication) and strains of *E. coli* used in *C. elegans* experiments lack the O-antigen needed for invasive pathogenesis (Browning et al., 2013). Through increased contact with intestinal cells, accumulation in the intestine may enhance other mechanisms by which *E. coli* are pathogenic, thus accelerating a cycle of functional loss in the aging intestine.

Second, we uncoupled the effects of *E. coli* growth from the *E. coli* activity that accelerates aging in *C. elegans*. Though SMX does not slow *E. coli* growth, we suggest it prevents a process that is also prevented by kanamycin. This process might be a factor that is induced by high folate levels and requires kanamycin-sensitive translation. The *E. coli* screen showed that lifespan is increased by only a few mutations, while many mutations slow *E. coli* growth without extending *C. elegans* lifespan. Thus, an alternative to the growth-dependent model is that a specific *E. coli* activity shortens lifespan, and this activity is blocked by treatment with kanamycin and other antibiotics.

How *E. coli* Influences *C. elegans* Aging

The comparison with kanamycin suggests that inhibiting folate synthesis removes a pro-aging activity rather than producing an anti-aging activity. A common factor of *E. coli* genes isolated in the screen such as *pabA, pabB, rpoS, tatC, znuB*, and *ompA,* is that they reduce virulence when mutated in a wide range of pathogenic bacteria (Brown and Stocker, 1987; Dong and Schellhorn, 2010; Gabbianelli et al., 2011; Ochsner et al., 2002; Teng et al., 2006). Lab strains of *E. coli* used for *C. elegans* culture do not have known virulence factors, but the genes isolated in the screen might regulate other, as yet unknown, factors with a milder, long-term effect on their hosts. The decreased aversion to *E. coli* mutants identified in the screen, or to *E. coli* treated with SMX, is consistent with the removal of a toxin (Figure S3C) (Melo and Ruvkun, 2012). It is likely that some of the many peptides and compounds secreted by *E. coli* are toxic to *C. elegans*. These products may or may not influence lifespan. This study suggests that *E. coli* shorten *C. elegans* lifespan through a form of toxin-based virulence that is milder than observed with human pathogens, but may nevertheless be important for chronic disease and aging.

It is interesting to note that many *E. coli* genes and processes were not found to increase *C. elegans* lifespan robustly in the screen. Surprisingly, genes involved in ubiquinone synthesis or respiration were not found (Saiki et al., 2008), although the Keio *ubiG* mutant did not grow sufficiently to be included in the screen. The *E. coli* genes of unknown function, which constitute over a third of the mutant collection (Baba et al., 2006), were not screened and may be important for interactions with the host by, for example, synthesizing as yet uncharacterized toxins.

The Role of Bacterial Folate

Our work suggests that in bacteria, folate has functions beyond its role in biosynthetic one-carbon metabolism. Mutation of *pabA* or *pabB* attenuates virulence in invasive bacteria, and the accepted explanation is that these mutants cannot grow in mammalian cells that lack PABA (Brown and Stocker, 1987; Chimalapati et al., 2011). However, another explanation is that these mutants are less able to produce toxins. Likewise, sulfonamides, which are less effective than most antibiotics in stopping bacterial growth, may be effective by preventing toxicity rather than growth. RpoS is an *E. coli* sigma factor activated in stationary phase to coordinate a global stress response, which includes increased virulence. Of the hundreds of genes reported to be under RpoS transcriptional control, *pabA* in *E. coli* (Weber et al., 2005) and *pabB* in *Bacillus subtilis* (Eymann et al., 2002) have been implicated in microarray experiments. Thus, folate synthesis may be stimulated by RpoS activity.

Possible Implications for Human Aging and Disease

We have presented evidence that *E. coli* accelerates *C. elegans* aging independently of *E. coli* growth and *C. elegans* folate metabolism. If a similar relationship existed in the human gut, molecular characterization of this mechanism may uncover targets to

intervene in aging and chronic disease. Chronic conditions such as obesity and inflammatory bowel disease are characterized by a dysbiosis of the microbiota, leading to an overgrowth of gamma proteobacteria, such as *E. coli*. (Winter and Bäumler, 2014). Interestingly, small intestine bacterial overgrowth leads to increased levels of serum folate, originating from dominating opportunistic bacteria (Camilo et al., 1996; Lakhani et al., 2008). Dysbiosis and the consequent overabundance of gamma proteobacteria may be an important factor in aging (Clark et al., 2015). This study shows that bacteria folate synthesis can be targeted without compromising host folate status, which can be maintained by selective supplementation.

EXPERIMENTAL PROCEDURES

C. elegans Strains

N2 (wild-type), SS104 *glp-4(bn2)*, UF208 (wild-type), and UF209 *gcp-2.1(ok1004)*. See Supplemental Information for *gcp-2.1* analysis.

E. coli Strains

OP50 *ura* (Brenner, 1974), OP50-GFP (containing plasmid pFPV25.1) (Labrousse et al., 2000), BW21153 (Keio collection wild-type), and WT Kan: BW21153 pGreen 0029 (Kan+). Strains from Keio collection as listed (Table S2). Keio strains transformed with complementation plasmids (Tables S1 and S3).

Culture Conditions

NGM was prepared using high purity agar as described (Virk et al., 2012). As necessary, 25 μg/ml carbenicillin was added to maintain plasmid selection (OP50-GFP and complemented Keio mutants). Kanamycin was added after 24 hr of bacterial growth as described (Garigan et al., 2002). Folinic acid, folic acid, and MTX were from Schircks Laboratories. For the defined media, peptone was replaced by purified amino acids (Supplemental Information) and 0.2× trace metals (Studier, 2005). Calcium chloride was omitted as it is in the trace metals.

Lifespan Analysis

Survival analyses were performed as described (Virk et al., 2012). Worms were cultured at 15°C and shifted to 25°C at the L3 stage. At 25°C, *E. coli* metabolism is more active than at lower temperatures (Virk et al., 2012). At the L4/young adult stage, animals were placed on bacteria under the experimental conditions. All lifespan data is in Table S1. Statistical significance was determined using log rank and Wilcoxon tests of the Kaplan-Meier survival model.

GFP Bacterial Accumulation Assay

Worms were prepared and set up as for lifespan analysis using OP50-GFP. From day 5, using a Leica M165 FL stereomicroscope with a GFP2 filter with a 510 nm + long pass emission spectrum, individual animals were scored every 2 days for survival and as having no accumulation, partial accumulation, or full accumulation (Figure S1).

Motility Assay

Animals were prepared as for lifespan analysis as described, with 16 plates per condition used and ten worms per plate. Animals were classified as described (Herndon et al., 2002).

C. elegans Growth/Body Size Analysis

Animals were imaged after 2 days of 25°C growth after a synchronized egg lay. Images were analyzed for body length as described in Supplemental Information.

Screening Method

See Supplemental Information.

Gene Complementation of Keio Mutants

The relevant genomic region was PCR amplified from BW21153 using primers with restriction sites (Table S3), cloned into the low copy pMMB67EH Amp^R plasmid (Virk et al., 2012), and transformed into the relevant mutant strain. The plasmid was used as a control.

Measuring *E. coli* Lawn Growth

To quantify bacteria in bacterial lawns, 2 ml of M9 was added to a 6 cm plate. The lawn was scraped off with a glass scraper. The liquid was then removed to a fresh tube. The total volume of the removed liquid was multiplied by the OD_{600} after a 5-fold dilution to generate a relative measure of bacteria.

Aversion Assay

Aversion was measured as number of worms off the lawn/total number of worms as described in Melo and Ruvkun (2012).

SUPPLEMENTAL INFORMATION

Supplemental Information includes Supplemental Experimental Procedures, four figures, and three tables and can be found with this article online at http://dx.doi.org/10.1016/j.celrep.2016.01.051.

AUTHOR CONTRIBUTIONS

Conceptualization; Supervision; and Funding Acquisition, D.W.; Methodology, D.W., B.V., J.J., C.A.M., A.R., N.C., and N.H.; Investigation, B.V., J.J., C.A.M., A.R., J.L., Y.L., M.C., and N.C.; Formal Analysis, B.V., C.A.M., J.J., D.W., and S.A.R.; and Writing, D.W., C.A.M., B.V., J.J., and S.A.R.

ACKNOWLEDGMENTS

We thank Sushmita Maitra, Gonçalo Correia, Liping Zhao, and Ian Henderson for useful discussions and Kasia Zmarzly, Emily Cardew, and Inna Feyst for technical assistance. We thank the *C. elegans* Genetics Center, the *C. elegans* Knockout Consortium, and NBRP-E.coli at NIG for strains. This work was supported by BBSRC studentships (B.V., C.A.M., and N.H.) and a BBSRC Award (BB/H01974X/1). M.C. was supported by IAESTE and the Durham Biophysical Sciences Institute.

Received: August 3, 2015
Revised: November 18, 2015
Accepted: January 14, 2016
Published: February 11, 2016

REFERENCES

Asrar, F.M., and O'Connor, D.L. (2005). Bacterially synthesized folate and supplemental folic acid are absorbed across the large intestine of piglets. J. Nutr. Biochem. *16*, 587–593.

Baba, T., Ara, T., Hasegawa, M., Takai, Y., Okumura, Y., Baba, M., Datsenko, K.A., Tomita, M., Wanner, B.L., and Mori, H. (2006). Construction of Escherichia coli K-12 in-frame, single-gene knockout mutants: the Keio collection. Mol. Syst. Biol. *2*, 2006 0008.

Bailey, L.B., and Gregory, J.F., 3rd. (1999). Folate metabolism and requirements. J. Nutr. *129*, 779–782.

Balamurugan, K., Ashokkumar, B., Moussaif, M., Sze, J.Y., and Said, H.M. (2007). Cloning and functional characterization of a folate transporter from the nematode Caenorhabditis elegans. Am. J. Physiol. Cell Physiol. *293*, C670–C681.

Battesti, A., Majdalani, N., and Gottesman, S. (2011). The RpoS-mediated general stress response in Escherichia coli. Annu. Rev. Microbiol. *65*, 189–213.

Brenner, S. (1974). The genetics of Caenorhabditis elegans. Genetics *77*, 71–94.

Brown, R.F., and Stocker, B.A. (1987). Salmonella typhi 205aTy, a strain with two attenuating auxotrophic characters, for use in laboratory teaching. Infect. Immun. *55*, 892–898.

Browning, D.F., Wells, T.J., França, F.L., Morris, F.C., Sevastsyanovich, Y.R., Bryant, J.A., Johnson, M.D., Lund, P.A., Cunningham, A.F., Hobman, J.L., et al. (2013). Laboratory adapted Escherichia coli K-12 becomes a pathogen of Caenorhabditis elegans upon restoration of O antigen biosynthesis. Mol. Microbiol. *87*, 939–950.

Cabreiro, F., Au, C., Leung, K.Y., Vergara-Irigaray, N., Cochemé, H.M., Noori, T., Weinkove, D., Schuster, E., Greene, N.D., and Gems, D. (2013). Metformin retards aging in C. elegans by altering microbial folate and methionine metabolism. Cell *153*, 228–239.

Camilo, E., Zimmerman, J., Mason, J.B., Golner, B., Russell, R., Selhub, J., and Rosenberg, I.H. (1996). Folate synthesized by bacteria in the human upper small intestine is assimilated by the host. Gastroenterology *110*, 991–998.

Chimalapati, S., Cohen, J., Camberlein, E., Durmort, C., Baxendale, H., de Vogel, C., van Belkum, A., and Brown, J.S. (2011). Infection with conditionally virulent Streptococcus pneumoniae Δpab strains induces antibody to conserved protein antigens but does not protect against systemic infection with heterologous strains. Infect. Immun. *79*, 4965–4976.

Clark, R.I., Salazar, A., Yamada, R., Fitz-Gibbon, S., Morselli, M., Alcaraz, J., Rana, A., Rera, M., Pellegrini, M., Ja, W.W., and Walker, D.W. (2015). Distinct shifts in microbiota composition during Drosophila aging impair intestinal function and drive mortality. Cell Rep. *12*, 1656–1667.

Collins, J.J., Huang, C., Hughes, S., and Kornfeld, K. (2008). The measurement and analysis of age-related changes in Caenorhabditis elegans. WormBook *24*, 1–21.

Dong, T., and Schellhorn, H.E. (2010). Role of RpoS in virulence of pathogens. Infect. Immun. *78*, 887–897.

Eymann, C., Homuth, G., Scharf, C., and Hecker, M. (2002). Bacillus subtilis functional genomics: global characterization of the stringent response by proteome and transcriptome analysis. J. Bacteriol. *184*, 2500–2520.

Gabbianelli, R., Scotti, R., Ammendola, S., Petrarca, P., Nicolini, L., and Battistoni, A. (2011). Role of ZnuABC and ZinT in Escherichia coli O157:H7 zinc acquisition and interaction with epithelial cells. BMC Microbiol. *11*, 36.

Garigan, D., Hsu, A.L., Fraser, A.G., Kamath, R.S., Ahringer, J., and Kenyon, C. (2002). Genetic analysis of tissue aging in Caenorhabditis elegans: a role for heat-shock factor and bacterial proliferation. Genetics *161*, 1101–1112.

Gems, D., and Riddle, D.L. (2000). Genetic, behavioral and environmental determinants of male longevity in Caenorhabditis elegans. Genetics *154*, 1597–1610.

Gomez, F., Monsalve, G.C., Tse, V., Saiki, R., Weng, E., Lee, L., Srinivasan, C., Frand, A.R., and Clarke, C.F. (2012). Delayed accumulation of intestinal coliform bacteria enhances life span and stress resistance in Caenorhabditis elegans fed respiratory deficient E. coli. BMC Microbiol. *12*, 300.

Grandison, R.C., Piper, M.D., and Partridge, L. (2009). Amino-acid imbalance explains extension of lifespan by dietary restriction in *Drosophila*. Nature *462*, 1061–1064.

Green, J.M., Nichols, B.P., and Matthews, R.G. (1996). Folate biosynthesis, reduction and polyglutamylation. In Escherichia coli and Salmonella, F.C. Neidhardt, ed. (ASM Press), pp. 665–673.

Greer, E.L., and Brunet, A. (2009). Different dietary restriction regimens extend lifespan by both independent and overlapping genetic pathways in C. elegans. Aging Cell *8*, 113–127.

Halsted, C.H., Ling, E.H., Luthi-Carter, R., Villanueva, J.A., Gardner, J.M., and Coyle, J.T. (1998). Folylpoly-gamma-glutamate carboxypeptidase from pig jejunum. Molecular characterization and relation to glutamate carboxypeptidase II. J. Biol. Chem. *273*, 20417–20424.

Hansen, M., Hsu, A.L., Dillin, A., and Kenyon, C. (2005). New genes tied to endocrine, metabolic, and dietary regulation of lifespan from a Caenorhabditis elegans genomic RNAi screen. PLoS Genet. *1*, 119–128.

Heintz, C., and Mair, W. (2014). You are what you host: microbiome modulation of the aging process. Cell *156*, 408–411.

Herndon, L.A., Schmeissner, P.J., Dudaronek, J.M., Brown, P.A., Listner, K.M., Sakano, Y., Paupard, M.C., Hall, D.H., and Driscoll, M. (2002). Stochastic and genetic factors influence tissue-specific decline in ageing C. elegans. Nature *419*, 808–814.

Hussein, M.J., Green, J.M., and Nichols, B.P. (1998). Characterization of mutations that allow p-aminobenzoyl-glutamate utilization by Escherichia coli. J. Bacteriol. *180*, 6260–6268.

Kwon, Y.K., Lu, W., Melamud, E., Khanam, N., Bognar, A., and Rabinowitz, J.D. (2008). A domino effect in antifolate drug action in Escherichia coli. Nat. Chem. Biol. *4*, 602–608.

Labrousse, A., Chauvet, S., Couillault, C., Kurz, C.L., and Ewbank, J.J. (2000). Caenorhabditis elegans is a model host for Salmonella typhimurium. Curr. Biol. *10*, 1543–1545.

Lakhani, S.V., Shah, H.N., Alexander, K., Finelli, F.C., Kirkpatrick, J.R., and Koch, T.R. (2008). Small intestinal bacterial overgrowth and thiamine deficiency after Roux-en-Y gastric bypass surgery in obese patients. Nutr. Res. *28*, 293–298.

Lakoff, A., Fazili, Z., Aufreiter, S., Pfeiffer, C.M., Connolly, B., Gregory, J.F., 3rd, Pencharz, P.B., and O'Connor, D.L. (2014). Folate is absorbed across the human colon: evidence by using enteric-coated caplets containing 13C-labeled [6S]-5-formyltetrahydrofolate. Am. J. Clin. Nutr. *100*, 1278–1286.

Lee, D., Hwang, W., Artan, M., Jeong, D.E., and Lee, S.J. (2015). Effects of nutritional components on aging. Aging Cell *14*, 8–16.

Lewis, J.A., and Fleming, J.T. (1995). Basic culture methods. Methods Cell Biol. *48*, 3–29.

Lozupone, C.A., Stombaugh, J.I., Gordon, J.I., Jansson, J.K., and Knight, R. (2012). Diversity, stability and resilience of the human gut microbiota. Nature *489*, 220–230.

Mair, W., and Dillin, A. (2008). Aging and survival: the genetics of life span extension by dietary restriction. Annu. Rev. Biochem. *77*, 727–754.

McGee, M.D., Weber, D., Day, N., Vitelli, C., Crippen, D., Herndon, L.A., Hall, D.H., and Melov, S. (2011). Loss of intestinal nuclei and intestinal integrity in aging C. elegans. Aging Cell *10*, 699–710.

Mello, C.C., Kramer, J.M., Stinchcomb, D., and Ambros, V. (1991). Efficient gene transfer in C.elegans: extrachromosomal maintenance and integration of transforming sequences. EMBO J. *10*, 3959–3970.

Melo, J.A., and Ruvkun, G. (2012). Inactivation of conserved C. elegans genes engages pathogen- and xenobiotic-associated defenses. Cell *149*, 452–466.

Ochsner, U.A., Snyder, A., Vasil, A.I., and Vasil, M.L. (2002). Effects of the twin-arginine translocase on secretion of virulence factors, stress response, and pathogenesis. Proc. Natl. Acad. Sci. USA *99*, 8312–8317.

Patzer, S.I., and Hantke, K. (1998). The ZnuABC high-affinity zinc uptake system and its regulator Zur in Escherichia coli. Mol. Microbiol. *28*, 1199–1210.

Portal-Celhay, C., Bradley, E.R., and Blaser, M.J. (2012). Control of intestinal bacterial proliferation in regulation of lifespan in Caenorhabditis elegans. BMC Microbiol. *12*, 49.

Rizza, W., Veronese, N., and Fontana, L. (2014). What are the roles of calorie restriction and diet quality in promoting healthy longevity? Ageing Res. Rev. *13*, 38–45.

Saiki, R., Lunceford, A.L., Bixler, T., Dang, P., Lee, W., Furukawa, S., Larsen, P.L., and Clarke, C.F. (2008). Altered bacterial metabolism, not coenzyme Q content, is responsible for the lifespan extension in Caenorhabditis elegans fed an Escherichia coli diet lacking coenzyme Q. Aging Cell *7*, 291–304.

Sanchez-Roman, I., and Barja, G. (2013). Regulation of longevity and oxidative stress by nutritional interventions: role of methionine restriction. Exp. Gerontol. *48*, 1030–1042.

Smith, S.G., Mahon, V., Lambert, M.A., and Fagan, R.P. (2007). A molecular Swiss army knife: OmpA structure, function and expression. FEMS Microbiol. Lett. *273*, 1–11.

Stanley, N.R., Findlay, K., Berks, B.C., and Palmer, T. (2001). Escherichia coli strains blocked in Tat-dependent protein export exhibit pleiotropic defects in the cell envelope. J. Bacteriol. *183*, 139–144.

Studier, F.W. (2005). Protein production by auto-induction in high density shaking cultures. Protein Expr. Purif. *41*, 207–234.

Teng, C.H., Xie, Y., Shin, S., Di Cello, F., Paul-Satyaseela, M., Cai, M., and Kim, K.S. (2006). Effects of ompA deletion on expression of type 1 fimbriae in Escherichia coli K1 strain RS218 and on the association of E. coli with human brain microvascular endothelial cells. Infect. Immun. *74*, 5609–5616.

Virk, B., Correia, G., Dixon, D.P., Feyst, I., Jia, J., Oberleitner, N., Briggs, Z., Hodge, E., Edwards, R., Ward, J., et al. (2012). Excessive folate synthesis limits lifespan in the C. elegans: E. coli aging model. BMC Biol. *10*, 67.

Watson, E., MacNeil, L.T., Ritter, A.D., Yilmaz, L.S., Rosebrock, A.P., Caudy, A.A., and Walhout, A.J. (2014). Interspecies systems biology uncovers metabolites affecting C. elegans gene expression and life history traits. Cell *156*, 759–770.

Weber, H., Polen, T., Heuveling, J., Wendisch, V.F., and Hengge, R. (2005). Genome-wide analysis of the general stress response network in Escherichia coli: sigmaS-dependent genes, promoters, and sigma factor selectivity. J. Bacteriol. *187*, 1591–1603.

Weinkove, D. (2015). Model super-organisms: Can the biochemical genetics of E. coli help us understand aging? Biochemist (Lond.) *37*, 12–15.

Winter, S.E., and Bäumler, A.J. (2014). Dysbiosis in the inflamed intestine: chance favors the prepared microbe. Gut Microbes *5*, 71–73.

Cell Reports, Volume *14*

Supplemental Information

Folate Acts in *E. coli* to Accelerate *C. elegans* Aging Independently of Bacterial Biosynthesis

Bhupinder Virk, Jie Jia, Claire A. Maynard, Adelaide Raimundo, Jolien Lefebvre, Shane A. Richards, Natalia Chetina, Yen Liang, Noel Helliwell, Marta Cipinska, and David Weinkove

Supplemental Information

Figure S1

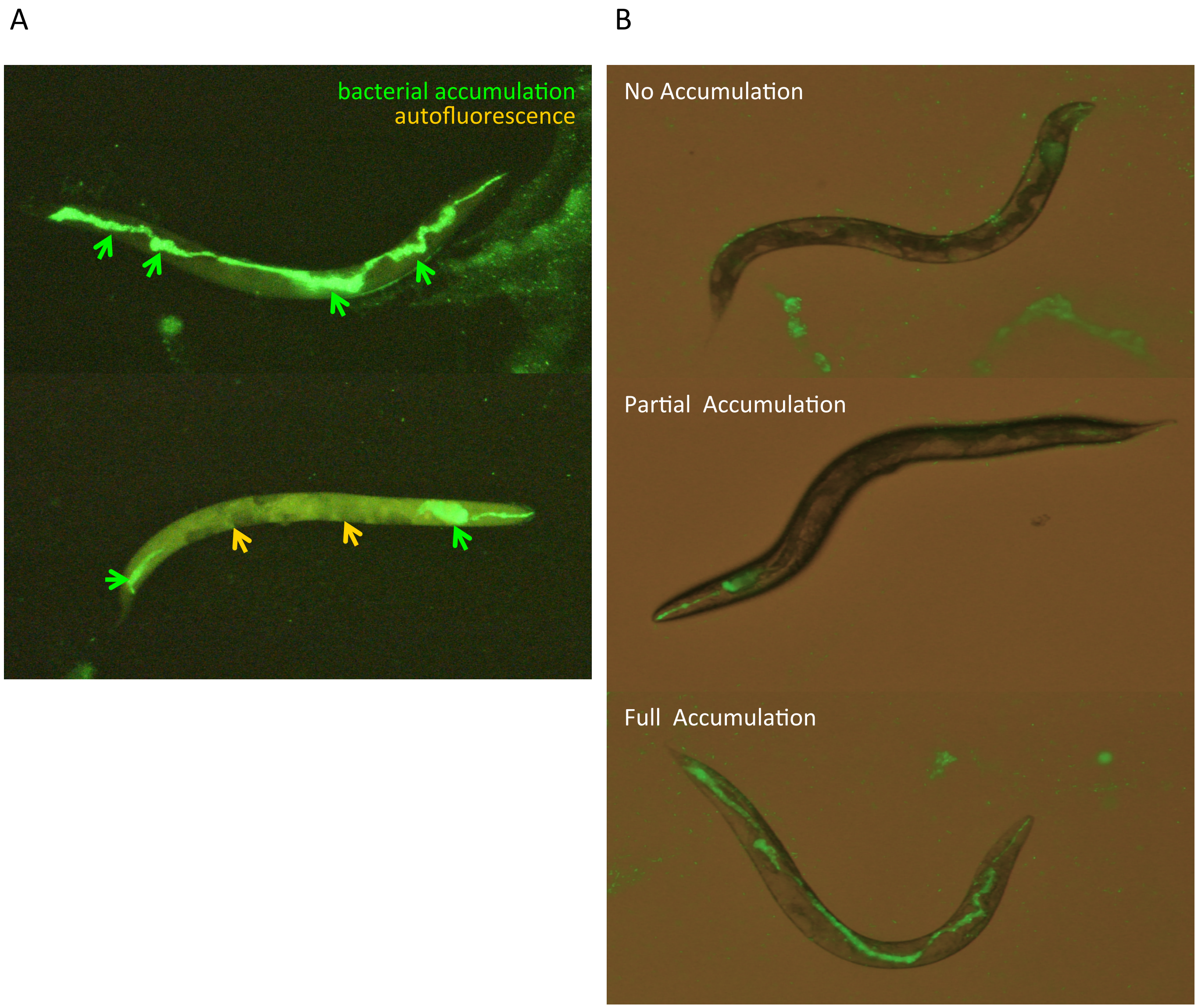

Figure S1. Distinguishing intestinal accumulation of *E. coli* OP50-GFP. Related to Figure 3. A) Representative images of single worms (observed using a long-pass green filter) illustrating the distinction between gut auto-fluorescence (yellow-green, yellow arrows) and GFP-expressing *E. coli* (bright green, green arrows). B) Representative images of single worms as classified according to extent of colonization by GFP-expressing *E. coli.*

Figure S2

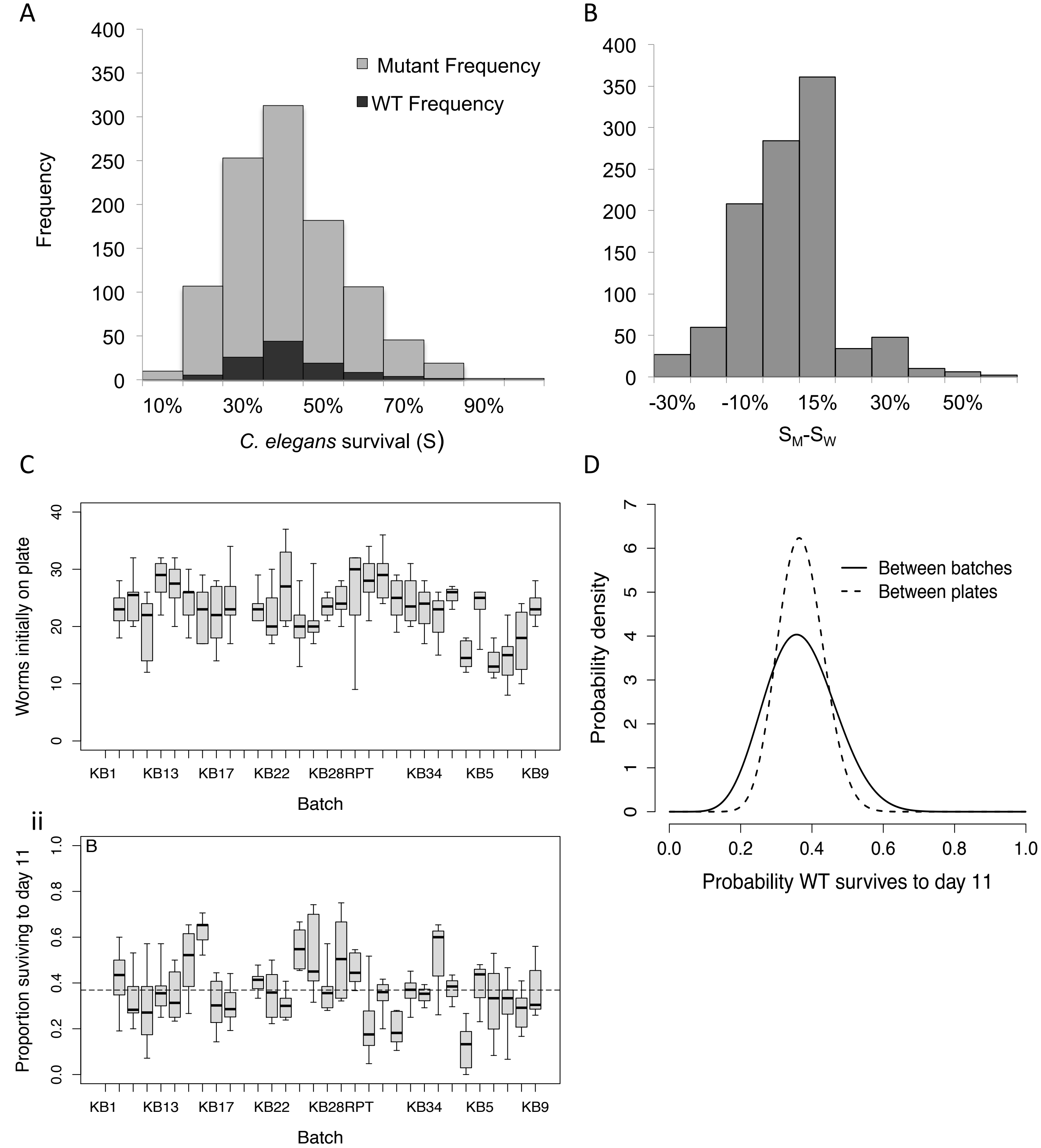

Figure S2. Estimating batch and plate variation on worm survival. Related to Figure 4. A) Histogram comparing the distribution in *C. elegans* survival in mutant and wild type strains. B) Normalizing the distribution to account for batch-to-batch variation: mutant survival (S_M) – wild type survival in the same batch (S_W). C) i) Variation in the number of WT worms scored per plate across batches ii) and the proportion of worms that survived in each plate. The dashed line indicates the maximum-likelihood fit for the overall survival fraction, *p*. All whiskers indicate the most extreme values. D) Best fitting beta distributions describing the variation in mean WT survival fraction to day 11 among plates (solid line), and variation in mean survival fraction between plates within a batch (dashed line). Here, we have depicted among plate variation for a batch having the overall mean survival, *p*. See Supplemental Experimental Procedures.

Supplemental Tables

(Excel Files)

Table S1. Lifespan summaries. **Related to Figures 2 and 4**. Contains conditions, numbers and relevant statistical analysis for all lifespan experiments in the study.

Table S2 Keio collection strains included in the screen. **Related to Figure 4**. A) Strains tested and their survival in the first round. B) Strains excluded because they did not grow on NGM plates.

Table S3 Strains selected for 2nd, 3rd and 4th rounds of the screen and the results. **Related to Figure 4.** Includes primers used to make complementation plasmids and the corresponding strains. Strains in red were added to 2nd and 3rd rounds because they were of interest.

Figure S3

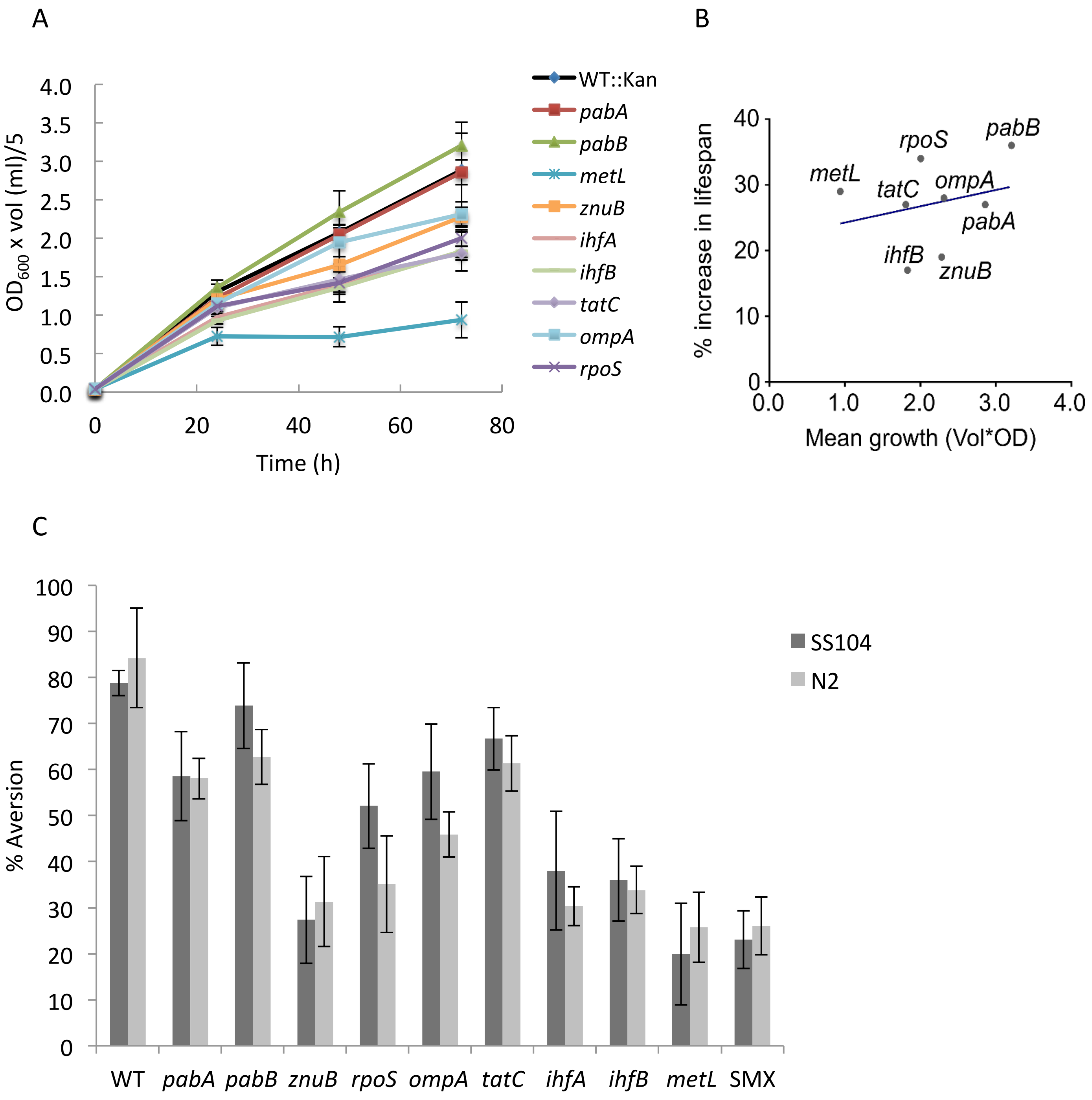

Figure S3. **Analysis of the 9 mutants isolated in the screen. Related to Figure 4.** A) Growth on NGM plates over 72 hours is significantly lower than wild type apart from *pabA* (no statistical difference), and *pabB* (significantly higher). See Experimental Procedures. Error bars represent standard deviation. B) Positive but not statistically significant correlation between bacterial growth on plates (A) and *C. elegans* lifespan increase for positive mutants from screen (Table S3). $R^2 = 0.06$, $P = 0.53$. C) Aversion of *C. elegans* strains to the bacterial lawn is decreased with the isolated mutants or with SMX. Error bars represent standard deviation. Aversion was statistically lower than control ($P < 0.01$) apart from on deletions of *pabB*, *ompA*, *tatC*, for which aversion is statistically lower only when data for N2 and SS104 are combined.

Figure S4

A

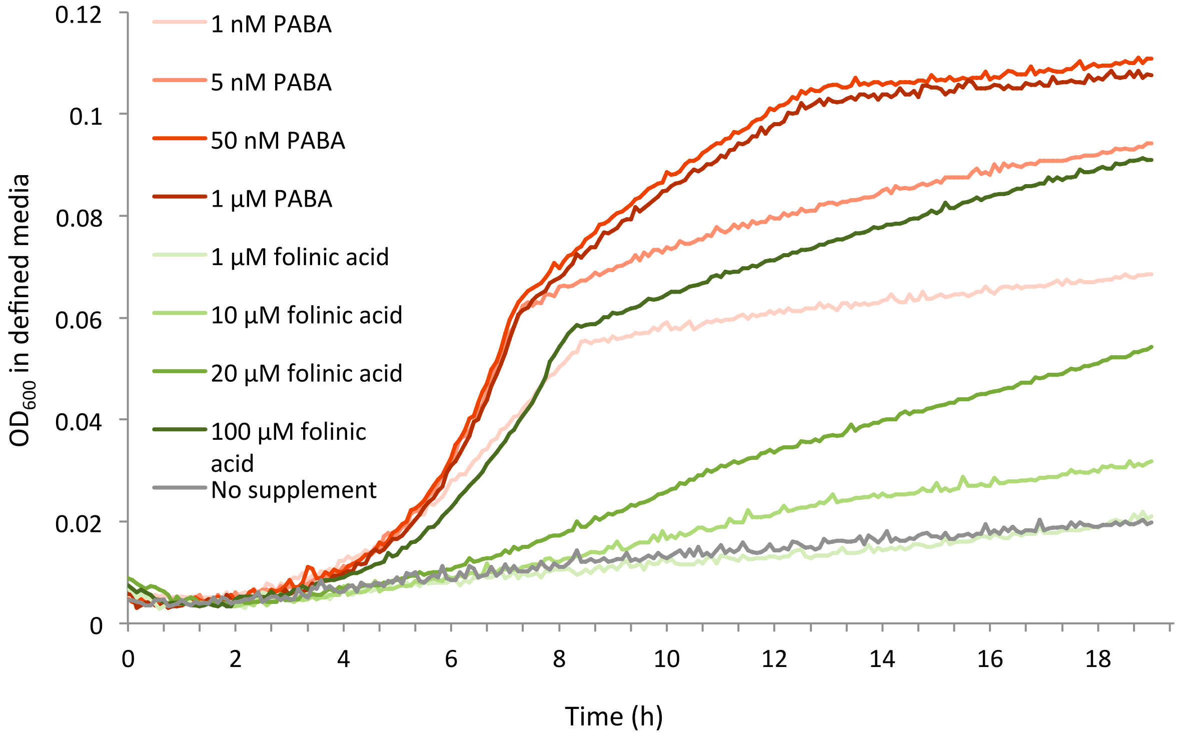

B

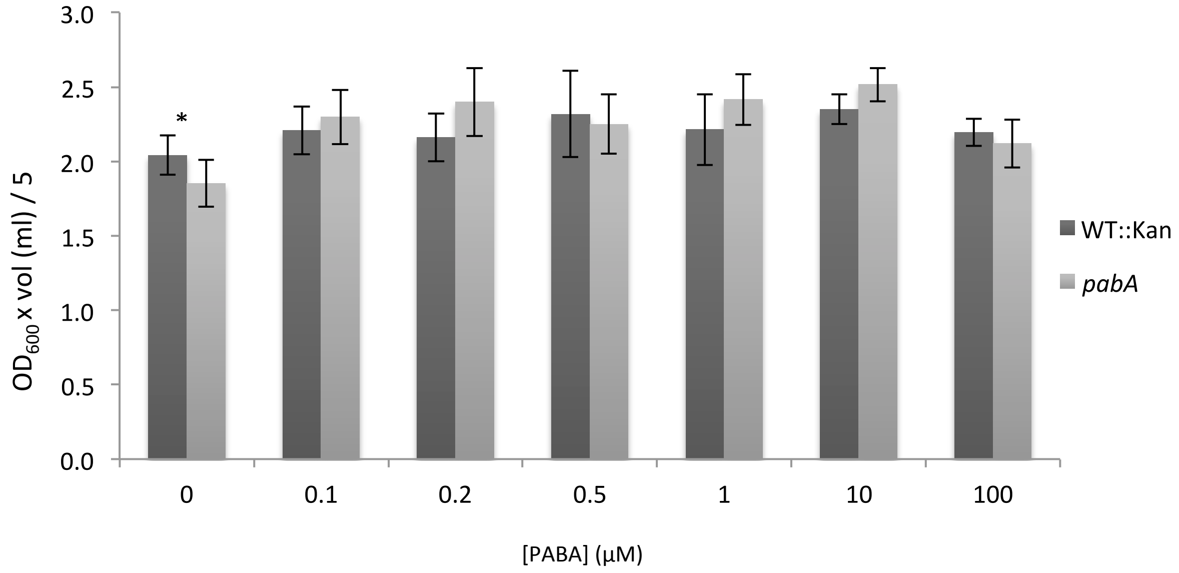

Figure S4. Growth of the *pabA* mutant. Related to Figure 4C and Figure 1. A) Growth curve of the *pabA* mutant in liquid defined media (Experimental Procedures) shows that 50 nM PABA is sufficient to restore bacterial growth. Folinic acid cannot completely restore growth at even at 100 µM. B) Growth on defined media plates with various concentrations of PABA after 72 hours, comparing *pabA* with WT under conditions used for Figure 4C. See Experimental Procedures. Error bars represent standard deviation. * p = < 0.01 for growth on *pabA* being less than growth on WT Kan.

Supplemental Experimental Procedures

GCP-2.1 characterization and analysis. Related to Figure 1

Three predicted proteins in *C. elegans* are encoded by R57.1 (named *gcp-2.1*), C35C5.2 (named *gcp-2.2*) and C35C5.11 (named *gcp-2.3*). All show similarity to mammalian GCPII amino acid sequences. The *gcp-2.1*(*ok1004*) deletion mutant from the *C. elegans* knockout consortium was outcrossed 3 times using N2 to make UF209 *gcp-2.1(ok1004).* The wild type sibling strain UF208 was used as the control. To make the *gcp-2.1* genomic transgene, a genomic fragment containing the predicted *gcp-2.1* gene was amplified using the primers R57gen_5: CTTAGGTTGGATCTCGTTGCTTGC and R57gen_3: TGTGTGGAAAGTGTGGTGAAGC using N2 genomic DNA as a template. 10 ng/µl of the PCR fragment with 90 ng/µl of marker plasmid *gpb-2*::*GFP* (van der Linden et al., 2001) was injected into UF209 *gcp-2.1(ok1004)* worms. A line transmitting the transgene mosaically, as assessed by GFP expression, was isolated (UF215 *gcp-2.1(ok1004) gqEx37[gcp-2.1, Pgbp-2::GFP].*). The transgene rescued the phenotypes of the *gcp-2.1* mutant.

Image analysis for worm growth. Related to Figure 1

Plates were imaged at 4.0x magnification using a Leica M165 FL stereomicroscope. The images were opened in ImageJ (Schneider et al., 2012) and the zoom function applied so that each image was 150% its original size, to enable more accurate measurement. The freehand line tool was then used to trace along the side of the body of each worm and the resulting line measured. To minimize bias, animals were selected for measurement randomly, using a grid overlay and then a random number generator to specify a grid reference. All animals in this specified square were then measured until a total of 30 worms had been measured for each condition. If an animal occupied two or more squares it was not measured.

Screening method. Related to Figure 4

Temperature sensitive sterile *glp-4(bn2)* worms were used in the screen. They were maintained at 15°C on OP50 and partly synchronized with an overnight timed egg lay. Three days later they were shifted to 25°C and then on the following day L4 worms were transferred to plates containing the bacterial strains to be

screened (Table S2A). Strains reported by Baba *et al.* to grow poorly in LB (<0.4 OD after 22 hours) and 15 strains that grew noticeably poorly on NGM plates were excluded (Table S2B). The screen plates were seeded 48 hours beforehand and left to grow at room temperature. The plates contained kanamycin (20 μg/ml) to kill the OP50. OP50-GFP was used to monitor bacterial persistence. For each strain at least 25 worms per plate were placed on at least 3 plates. For each batch, which consisted of 30-80 strains, 9 plates of 25+ worms were set up on a wild type BW21153 transformed with the Kan-containing plasmid pGreen 0029 (WT Kan). Plates were maintained at 25°C and survival was scored at either 11 or 12 days after the first day of adulthood. WT Kan plates were scored at both day 11 and 12. Survival was scored as number of alive worms/total number of worms. Strains that showed at least 15% increased survival (S_M) compared to the wild type control (S_W) were tested a second round of the screen (Table S3). Strains that passed this round, along with three other strains of interest (Table S3), were verified by PCR and tested in a full lifespan analysis using over 130 worms per strain in which survival was scored every two or three days. (Table S3). For strains that increased lifespan significantly in this third round, we complemented the missing gene with a wild type copy carried on a low copy plasmid (Table S3). The mutant *ihfA*, due to difficulties cloning the complementation construct, was confirmed by testing the "even" strain from the Keio collection (Table S1).

Estimating batch and plate variation on worm survival. Related to Figure 4A and Figure S2

First, we quantified the degree of variation in wild type (WT) survival both between batches and between plates within a batch. For simplicity we restricted our analysis to worms that were scored on day 11 (we find similar results when we examine worms scored on day 12). Let B be the number of batches performed and let P_i be the number of WT plates scored on day 11 in batch i. In total, we estimated variation in WT survival by day 11 from 209 plates distributed across B = 30 batches. The number of worms initially on a plate varied from 8 to 37 (mean = 22.66 worms), and the number of plates per batch varied from 3 to 12 (mean = 6.97 plates) (Figure S2Ci). Suppose the variation in the mean WT survival among batches has a beta-distribution with mean $\bar{p}$ and variance $\bar{p}(1-\bar{p})\emptyset_b/(1+\emptyset_b)$ (see (Richards, 2008) for details). Also, suppose that variation in mean survival among plates within a batch also has a beta-distribution with variance parameter $\emptyset_p$. These assumptions seem reasonable when inspecting the raw data describing the

between batch and between plate variation in survival fractions (Figure S2Cii). Under these assumptions, if n_{ij} of the N_{ij} worms on plate j in batch i survive to day 11, then the likelihood of the model, described by the set of parameters: $\boldsymbol{q}$ = $\{\bar{p}, \emptyset_\mathrm{b}, \emptyset_\mathrm{p}\}$, is given by

$$L(\boldsymbol{\theta}|\mathrm{data}) = \prod_{i=1}^{B} \int_{p=0}^{1} f_\mathrm{b}(p|\bar{p}, \emptyset_\mathrm{b}) \prod_{j=1}^{P_i} f_\mathrm{bb}\big(n_{ij}|N_{ij}, p, \emptyset_\mathrm{p}\big)\, dp, \quad (1)$$

where f_b and f_bb are the probability density functions for the beta and the beta-binomial distributions (see (Richards, 2008) for details). Likelihood ratio tests (LRTs) were used to evaluate if $\emptyset_\mathrm{b}$ or $\emptyset_\mathrm{p}$ were significantly different from zero (i.e. to test if there is no between batch or between plate variation). We found strong evidence of variation in WT survival between plates (LRT, G_1 = 52.6, P < 0.001) and variation in survival between plates within a batch (LRT, G_1 = 11.7, P = 0.001). Our best-fitting model was described by $\boldsymbol{q}$ = $\{\bar{p}$ = 0.369, $\emptyset_\mathrm{b}$ = 0.042, $\emptyset_\mathrm{p}$ = 0.017$\}$, which, as expected, predicts between batch variation being greater than between plate variation (Figure S2D).

Defined medium amino acid mix

The amino acid mix was designed using the composition of soy peptone. Together with trace metals, the mix was used to replace peptone in NGM (See Experimental Procedures for details). The concentration of the total mix was adjusted downwards so that growth of *E. coli* on defined medium agar plates was similar to that on peptone agar plates. Final concentrations (g/L): Alanine (1.419), Arginine (1.293), Aspartic acid (1.782), Cysteine (0.102), Glutamic acid (2.994), Glycine (3.414), Histidine (0.282), Isoleucine (0.468), Leucine (0.846), Lysine (0.924), Methionine (0.219), Phenylalanine (0.549), Proline (2.064), Serine (0.843), Threonine (0.534), Tryptophan (0.057), Tyrosine (0.318), Valine (0.627).

Supplemental References

Richards, S.A. (2008). Dealing with Overdispersed Count Data in Applied Ecology. Journal of Applied Ecology *45*, 218-227.

Schneider, C.A., Rasband, W.S., and Eliceiri, K.W. (2012). NIH Image to ImageJ: 25 years of image analysis. Nature Methods *9*, 671-675.

van der Linden, A.M., Simmer, F., Cuppen, E., and Plasterk, R.H. (2001). The G-protein beta-subunit GPB-2 in Caenorhabditis elegans regulates the G(o)alpha-G(q)alpha signaling network through interactions with the regulator of G-protein signaling proteins EGL-10 and EAT-16. Genetics *158*, 221-235.
